# Endometrial Vitamin D Signaling and Immune Escape in Recurrent Pregnancy Loss

**DOI:** 10.3390/cells15151405

**Published:** 2026-08-03

**Authors:** Charalampos Voros, Fotios Chatzinikolaou, Georgios Papadimas, Ioannis Papapanagiotou, Nektaria Zagorianakou, Ali Can Gunes, Aristotelis-Marios Koulakmanidis, Athanasios Karpouzos, Kyriakos Bananis, Charalampos Tsimpoukelis, Maria Anastasia Daskalaki, Christina-Maria Trakatelli, Stylianos Makrydimas, Nikolaos Thomakos, Panagiotis Antsaklis, Dimitrios Loutradis, George Daskalakis

**Affiliations:** 1 Department of Obstetrics and Gynecology, ‘Alexandra’ General Hospital, National and Kapodistrian University of Athens, 80 Vasilissis Sofias Avenue, 11528 Athens, Greece; 2 Laboratory of Forensic Medicine and Toxicology, School of Medicine, Aristotle University of Thessaloniki, 54124 Athens, Greece; 3 Athens Medical School, National and Kapodistrian University of Athens, 15772 Athens, Greece; 4 Scientific Laboratory for Innovative Technologies in Internal Medicine, Preventive Medicine and Overall Care, Department of Nursing, School of Health Sciences, University of Ioannina, 45500 Ioannina, Greece; 5 Department of Obstetrics and Gynecology, Faculty of Medicine, Hacettepe University, Ankara 06100, Turkey; 6 King’s College Hospitals NHS Foundation Trust, London SE5 9RS, UK; 7 3rd Department of Internal Medicine, Aristotle University of Thessaloniki, 54124 Thessaloniki, Greece; 8 Medical School, Aristotle University of Thessaloniki, 54124 Thessaloniki, Greece

**Keywords:** recurrent pregnancy loss, infertility, vitamin D, in vitro fertilization, implantation, mitochondrial dysfunction

## Abstract

**Highlights:**

**What are the main findings?**

- Endometrial CYP27B1 activates vitamin D locally, partly independent of circulating 25(OH)D levels, and this local signaling governs stromal cell differentiation and progesterone responsiveness.
- VDR disruption shifts decidual immunity toward Th17 dominance over Treg tolerance, and alters uterine NK-cell function, macrophage polarization, and trophoblast invasion.

**What are the implications of the main findings?**

- Local, tissue-level vitamin D signaling not just circulating levels may drive decidual dysfunction in recurrent pregnancy loss, with oxidative stress, mitochondrial dysfunction, and ferroptosis potentially amplifying this injury downstream.
- Serum vitamin D measurement alone cannot capture endometrial receptor dysfunction; endometrial VDR profiling and immunometabolic stratification are potential future directions, pending clinical validation.

**Abstract:**

Recurrent pregnancy loss (RPL) continues to be a significant challenge in reproductive medicine, particularly in women for whom standard examinations do not reveal a conclusive underlying reason. There is growing evidence that several instances may result from nuanced alterations in the endometrial milieu, particularly with decidualization and maternal–fetal immune tolerance. In recent years, vitamin D has gained recognition for its significance in early pregnancy, serving not only as a regulator of calcium metabolism but also as an active contributor to endometrial and immunological functions. The human endometrium exhibits the vitamin D receptor (VDR) and the enzyme CYP27B1, facilitating the local activation and signaling of vitamin D inside the uterine milieu. Experimental investigations have shown that vitamin D influences many processes critical for effective implantation and placentation, including stromal cell differentiation, cytokine equilibrium, trophoblast invasion, oxidative stress responses, and immune cell communication. Aberrant vitamin D signaling has been associated with heightened inflammatory activity, impaired decidual transformation, altered uterine natural killer cell functionality, and alteration of the Treg/Th17 equilibrium, all of which have been implicated in recurrent pregnancy loss. Concurrently, there is an increasing emphasis on the association between vitamin D and mitochondrial function as well as oxidative stress in decidual and endometrial cells. Interruption of these pathways may influence implantation and early embryonic development by impacting cellular metabolism and immunological control at the maternal–fetal interface. The clinical interest in vitamin D supplementation for women experiencing repeated reproductive failure is increasing; nevertheless, the existing results are conflicting, mostly due to the predominance of research focusing on circulating vitamin D levels rather than localized tissue-specific processes. Our review encapsulates new findings about the function of vitamin D in endometrial biology and reproductive immune regulation, emphasizing its involvement in decidualization, inflammatory signaling, oxidative stress, and maternal–fetal immunological tolerance in recurrent pregnancy loss. The potential ramifications for assisted reproduction and forthcoming tailored therapy techniques are also examined.

## 1. Introduction

Recurrent pregnancy loss (RPL) continues to be one of the most difficult conditions to manage in reproductive medicine, particularly in women for whom standard examinations do not reveal a definitive underlying reason [1]. Throughout this review, recurrent pregnancy loss (RPL) and recurrent miscarriage are used synonymously, consistent with current ESHRE and ASRM usage, and the term used in a given passage generally follows the terminology of the study being cited. Implantation failure refers specifically to failure of embryo attachment or early loss prior to clinical pregnancy recognition, most often discussed in the context of assisted reproduction, while reproductive failure is used as a broader umbrella term when the underlying mechanistic evidence spans both RPL and implantation failure without distinguishing between them. A fraction of miscarriages is attributable to chromosomal abnormalities, endocrine problems, thrombophilia, uterine pathology, and autoimmune illnesses. Still, after thorough assessment, several couples continue to receive a diagnosis of unexplained RPL. Over the last decade, there has been a growing emphasis on the maternal aspects of implantation, particularly the significance of the endometrium and the local immunological milieu during the first phases of pregnancy [2].

Implantation is an active process involving both the embryo and the uterine cavity, coordinating decidual transformation, immune adaptation, angiogenesis, cytokine signaling, and trophoblast invasion. A successful pregnancy requires the formation of a responsive and immunologically balanced endometrial environment that can sustain the semi-allogeneic embryo. Interruption of this process may result in flawed implantation, compromised placentation, or premature embryonic loss. Recurrent miscarriage is now seen as not solely an embryonic illness but also as a dysfunction of aberrant endometrial activity and compromised maternal–fetal immunological tolerance [3].

Vitamin D has lately garnered significant interest regarding its role in the regulation of the endometrium. In addition to its traditional function in calcium metabolism, vitamin D seems to be a significant immunological and cellular regulator in reproductive organs. The presence of the vitamin D receptor (VDR) and the enzyme CYP27B1 in the endometrium, decidua, trophoblast, and diverse immune cell populations supports the concept of localized vitamin D activation and signaling at the maternal–fetal interface. The results suggest that vitamin D may affect implantation and early pregnancy via tissue-specific autocrine and paracrine pathways, in addition to systemic endocrine action [4].

Simultaneously, increasing data suggests that oxidative stress and mitochondrial dysfunction may significantly contribute to reproductive failure. The excessive generation of reactive oxygen species (ROS) in the decidual milieu may interfere with cellular metabolism, inflammatory signaling, and trophoblast–endometrium communication during early pregnancy. Oxidative stress and antioxidant balance are increasingly recognized as important determinants of pregnancy outcome [5], and vitamin D is thought to intersect with these pathways, such that disrupted vitamin D signaling may contribute to immunological and metabolic abnormalities at the maternal–fetal interface [5].

Current data is uneven and sometimes difficult to interpret, despite growing therapeutic interest in vitamin D treatment for women experiencing repeated reproductive failure. The majority of existing research focus on blood vitamin D levels, but there is far less understanding of local vitamin D signaling in the endometrium and decidua [6]. Thus, clarifying the cellular processes that govern vitamin D’s modulation of immunological tolerance and endometrial function may provide significant insights into the mechanisms behind recurrent pregnancy loss. This research encapsulates the existing knowledge about the function of vitamin D signaling within the endometrial and decidual milieu, emphasizing decidualization, immunological dysregulation, oxidative stress, mitochondrial dysfunction, and maternal–fetal tolerance in recurrent pregnancy loss. The potential translational implications for assisted reproduction and personalized reproductive medicine are examined [4].

## 2. Materials and Methods

We conducted a narrative literature analysis using the PubMed database to uncover publications examining the relationship between vitamin D transmission and recurrent pregnancy loss, endometrial receptivity, decidualization, maternal–fetal immunological tolerance, oxidative stress, and assisted reproduction. The literature was examined up to May 2026.

The search phrases were a blend of Medical Subject Headings (MeSH) and free-text terms related to vitamin D and reproductive immunology. The primary search terms included “vitamin D”, “vitamin D receptor”, “VDR”, “CYP27B1”, “recurrent pregnancy loss”, “recurrent miscarriage”, “endometrium”, “decidua”, “implantation failure”, “maternal–fetal interface”, “immune tolerance”, “uterine natural killer cells”, “Treg/Th17”, “oxidative stress”, “mitochondrial dysfunction”, “decidualization”, and “assisted reproduction”. Further studies were discovered by a manual examination of the reference lists of pertinent papers.

Studies were selected if they pertained to the molecular, immunological, cellular, or clinical dimensions of vitamin D communication in reproductive tissues, with particular focus on recurrent pregnancy loss and endometrial biology. Included were human research, translational studies, experimental studies, and mechanistic reviews pertinent to immune modulation, decidual function, inflammatory indicating, oxidative stress, and implantation biology. Recent investigations prioritizing tissue-specific processes in the endometrial and decidual milieu were conducted.

We omitted research that were unrelated to vitamin D regulation at the maternal–fetal interface or to reproductive biology. Titles and abstracts were assessed for relevance, and chosen papers underwent comprehensive evaluation. The extracted data concentrated on vitamin D receptor expression, local vitamin D metabolism, decidualization pathways, immune cell regulation, cytokine signals, oxidative stress responses, mitochondrial dysfunction, trophoblast invasion, and translational implications for recurrent pregnancy loss and assisted reproduction.

Only peer-reviewed publications in English were considered; conference abstracts and preprints were excluded to preserve consistency in reporting quality. Where studies reported conflicting findings, we prioritized human and clinical data over animal or in vitro evidence, and we have noted disagreement explicitly rather than resolving it in favor of a single interpretation. This is a narrative rather than a systematic review: no formal risk-of-bias assessment or quantitative synthesis was performed, study selection was not conducted independently by two reviewers, and the absence of a preregistered protocol means that publication bias and selective emphasis cannot be excluded. These limitations are acknowledged, and the conclusions drawn should be interpreted as a synthesis of the current conceptual landscape rather than a definitive evidence hierarchy.

Synthesis of the retrieved literature was organized thematically rather than chronologically or by study design, following the mechanistic structure of this review: vitamin D metabolism and receptor biology, decidualization and progesterone signaling, immune regulation, oxidative and mitochondrial pathways, and clinical/translational evidence. Within each theme, findings were grouped by the level of evidence available (mechanistic/experimental versus human observational or interventional), consistent with the evidence distinctions made throughout the manuscript.

## 3. Endometrial Vitamin D Signaling

### 3.1. Vitamin D Metabolism Within the Endometrium

Vitamin D transmission in the endometrium is becoming acknowledged as a locally controlled molecular mechanism, rather than just a downstream reaction to circulating blood vitamin D levels. The human endometrium exhibits the VDR and the enzymatic apparatus for intracellular vitamin D activation and degradation, hence endorsing the notion of tissue-specific autocrine and paracrine communication during implantation and early pregnancy [7]. Vitamin D is metabolized and locally acted upon by endometrial stromal cells, glandular epithelial cells, decidual cells, trophoblasts, and several immune cell types at the maternal–fetal interface [8].

CYP27B1 catalyzes the conversion of the physiologically inactive precursor 25-hydroxyvitamin D [25(OH)D] into the active metabolite 1,25-dihydroxyvitamin D3 [1,25(OH)2D3] inside cells. The presence of CYP27B1 in the endometrium suggests that vitamin D activation may occur locally in decidual and implantation-associated tissues, independent of renal metabolism [9]. The principal enzyme responsible for vitamin D catabolism, CYP24A1, is also expressed locally. This indicates a regulated network governing the activation and degradation of vitamin D within the uterine milieu.

This local activation capacity does not operate independently of systemic vitamin D status in an absolute sense. CYP27B1 requires 25(OH)D as substrate, so severe systemic deficiency would be expected to constrain local activation regardless of endometrial CYP27B1 expression levels. The relationship is better described as a partial uncoupling than a full independence: circulating 25(OH)D likely sets an upper bound on locally available substrate, while endometrial CYP27B1 and CYP24A1 activity, VDR expression, and downstream transcriptional responsiveness determine how efficiently that substrate is converted and used within the tissue itself. Whether this local–systemic relationship differs between women with recurrent pregnancy loss and fertile controls has not been directly examined in available studies, and this remains an open question rather than an established finding.

Upon activation, 1,25(OH)2D3 binds to the intracellular vitamin D receptor, which then heterodimerizes with the retinoid X receptor (RXR). The VDR–RXR complex migrates to the nucleus and interacts with vitamin D response elements (VDREs) in the promoter regions of target genes. This method allows vitamin D to regulate transcriptional programs related to immunological regulation, cell differentiation, oxidative stress responses, extracellular matrix remodeling, angiogenesis, and inflammatory activation. Several of these pathways are essential for implantation and decidual transformation [10].

The transcriptional activity associated with decidualization is evidently closely connected to vitamin D demonstration in endometrial stromal cells. Activation of VDR influences the expression of genes associated with progesterone responsiveness, stromal differentiation, and implantation competence, including prolactin, insulin-like growth factor binding protein-1 (IGFBP-1), HOXA10, and integrin-related pathways [4]. HOXA10 is essential for endometrial receptivity and embryo attachment. Aberrant expression of implantation-associated adhesion molecules has been associated with implantation failure and recurrent miscarriage [11].

### 3.2. Vitamin D Receptor Expression in Endometrial and Decidual Cells

The presence of the vitamin D receptor in reproductive organs substantiates the concept that vitamin D signaling has a direct role in implantation and the maintenance of early pregnancy, rather than functioning exclusively via systemic endocrine pathways. Receptor expression exhibits variability throughout the menstrual cycle, demonstrating heightened activity during the secretory phase and peri-implantation period, indicating a strong correlation between vitamin D responsiveness and endometrial receptivity. This temporal control is physically feasible, as implantation requires precisely coordinated interactions among decidual cells, trophoblasts, immunological mediators, and local inflammatory signals [12].

The VDR seems to be especially significant in the stromal compartment during decidual transition. During the luteal phase, endometrial stromal cells undergo significant reprogramming to adopt a specialized secretory and immunomodulatory phenotype that facilitates embryo implantation. The activation of VDR affects many transcriptional pathways associated with cellular differentiation, cytoskeletal rearrangement, extracellular matrix architecture, and progesterone responsiveness [4]. Consequently, impaired receptor communication might hinder the transition of the proliferative endometrium to a functioning decidua. Such changes may create a physiologically unstable implantation environment, characterized by insufficient trophoblast support, aberrant inflammatory activity, and impaired maternal adaption to pregnancy [13].

In addition to its influence on stromal differentiation, VDR communication seems to be closely associated with the regulation of genes relevant to implantation. Vitamin D-dependent transcriptional activity influences several molecules related to embryo attachment and interaction with the decidua, both directly and indirectly [14]. These include HOXA10, leukemia inhibitory factor (LIF), osteopontin, integrins, and other adhesion molecules critical for endometrial receptivity. The downregulation of these pathways may negatively impact embryo–endometrial interactions during the implantation window and might contribute to recurrent implantation failure and early miscarriage. The modified expression of implantation-related genes has been consistently reported in women experiencing recurrent reproductive failure, reinforcing the notion that impaired local vitamin D transmission may disrupt endometrial competence at many cellular levels [15].

Moreover, vitamin D receptor activity seems to be very pertinent in trophoblastic tissue. Successful placentation requires regulated infiltration of trophoblast cells into the decidua and spiral arteries. This process requires a balance of inflammatory announcing, extracellular matrix breakdown, angiogenesis, and immunological adaptability [16]. The activation of VDR affects many pathways pertinent to trophoblast migration and invasion, including matrix metalloproteinases, VEGF-associated indicating, and cytokine-mediated interactions between trophoblasts and decidual cells. Decreased receptor activation may impair trophoblastic invasion of maternal tissues, resulting in insufficient placental growth and unstable early pregnancy. Such problems are especially relevant in recurrent pregnancy loss, when faulty placentation and aberrant maternal vascular remodeling are increasingly acknowledged as contributory factors [17].

The relationship between VDR activity and immune control at the maternal–fetal interface also seems to be of equal significance. Implantation necessitates the creation of a regulated inflammatory milieu that may facilitate tissue remodeling while preserving immunological tolerance towards the semi-allogeneic embryo [18]. Various immune cell types, including uterine natural killer cells, macrophages, dendritic cells, and regulatory T-cells, exhibit functional vitamin D receptors involved in this process. The activation of VDR influences differentiation, cytokine synthesis, cellular maturation, and inflammatory responses in these populations. Consequently, diminished receptor instructing may lead to heightened inflammatory responses, augmented cytotoxicity, and reduced maternal immunological tolerance in early pregnancy [19].

Modifications in VDR activity have been linked in several studies to disruptions in the Treg/Th17 equilibrium, a prominent immunological anomaly in recurrent pregnancy loss. Vitamin D seems to inhibit pro-inflammatory Th17-mediated responses and promote the proliferation and stability of regulatory T-cell populations [20]. Decreased receptor activation may result in a more inflammatory decidual immunological milieu characterized by elevated production of IL-17, TNF-α, and IFN-γ. These modifications may hinder the invasion of the trophoblast and the immunological balance necessary for implantation and placental development. Chronic inflammatory activation in the decidua may potentially lead to the buildup of oxidative stress and localized tissue damage during early gestation [21].

The interaction with steroid hormone pathways complicates the control of the endometrium by vitamin D communication. The interaction between VDR activity and progesterone signaling is especially significant during the luteal phase and early pregnancy, as progesterone-induced decidualization is essential for maintaining implantation stability. Inadequate vitamin D signaling may impair progesterone responsiveness in decidual stromal cells, leading to persistent inflammatory activation despite adequate circulating hormone levels. Experimental studies indicate that vitamin D may modify the expression of progesterone-responsive genes associated with angiogenesis, immunological tolerance, and stromal cell survival [22].

### 3.3. Genomic and Non-Genomic Pathways of Vitamin D Communication

The endometrium exhibits a complex interaction of conventional genomic and fast non-genomic routes of vitamin D communication, which are essential for optimal implantation and early placental development. These transduction systems operate in conjunction with inflammatory cascades, steroid hormone pathways, mitochondrial control, cellular metabolism, and immunological adaptability at the maternal–fetal interface [23]. Vitamin D seems to influence endometrial stromal cells, decidual cells, trophoblasts, and local immune cell populations via many communications within cell pathways. This extensive biological activity indicates that changes in vitamin D responsiveness may influence implantation throughout several molecular pathways simultaneously, rather than through a single isolated mechanism [24].

#### 3.3.1. Genomic Vitamin D Signaling

The genomic route of vitamin D activation is defined by the hydroxylation of inert 25-hydroxyvitamin D to the physiologically active metabolite 1,25-dihydroxyvitamin D3, facilitated by the intracellular enzyme CYP27B1. Activated vitamin D interacts with the intracellular vitamin D receptor, inducing conformational changes that promote heterodimerization with the retinoid X receptor [25]. The VDR–RXR complex subsequently translocates to the nucleus, where it interacts with vitamin D response elements in the promoter regions of target genes. This approach would allow vitamin D to directly influence transcriptional programs related to decidual differentiation, inflammatory signaling, angiogenesis, oxidative stress response, extracellular matrix organization, cellular proliferation, and immunological tolerance effects that have been demonstrated for 1,25-dihydroxyvitamin D3-VDR signaling in other cellular systems, including regulation of oxidative stress, inflammatory, angiogenic, and extracellular matrix remodeling protein expression [26]. Such broad transcriptional control would plausibly carry particular significance during the peri-implantation phase, characterized by simultaneous rapid tissue remodeling and immunological response, though this reproductive-specific extension has not been directly demonstrated.

Numerous genes linked to implantation competence and endometrial receptivity seem to be significantly influenced by VDR-mediated transcriptional activity [12,14]. The activation of vitamin D mechanisms of signaling may modify the expression of HOXA10, leukemia inhibitory factor, osteopontin, integrins, VEGF, and several progesterone-responsive molecules. A significant number of these genes are directly implicated in embryo attachment, stromal cell differentiation, trophoblast invasion, and vascular remodeling during early placentation. Decreased activation of these pathways may impede embryo–endometrial communication and jeopardize implantation before the discovery of clinical pregnancy. Growing data indicates that even minor irregularities in implantation-related transcriptional networks may play a role in recurrent pregnancy loss and implantation failure, despite seemingly normal embryonic development [27].

Genomic vitamin D signaling has extensive immunomodulatory effects in decidual tissue. The activation of the VDR suppresses the transcription of many pro-inflammatory mediators by disrupting nuclear factor kappa B (NF-κB)-dependent inflammatory communication. Activation of vitamin D has been shown to downregulate the synthesis of IL-6, IL-17, TNF-α, and IFN-γ, while potentially upregulating the expression of anti-inflammatory mediators such as IL-10 and TGF-β [28]. Nevertheless, excessive inflammation may hinder trophoblast invasion and maternal immunological tolerance. Dysregulated inflammatory activation during the implantation window may be a primary mechanism connecting decreased vitamin D response with repeated reproductive failure [29].

#### 3.3.2. Non-Genomic Vitamin D Signaling

In addition to its transcriptional effects, vitamin D initiates fast non-genomic pathways of communication that may affect cellular activity within minutes. These mechanisms appear to be facilitated by membrane-associated VDR-related complexes and the activation of intracellular kinases rather than direct nuclear transcription. Numerous reproductive organs exhibit fast activation of phosphatidylinositol 3-kinase, AKT, mitogen-activated protein kinase, phospholipase C, calcium-dependent pathways, and protein kinase cascades. These pathways govern cellular survival, mitochondrial adaptability, oxidative stress responses, cytoskeletal architecture, intracellular calcium homeostasis, and inflammatory signal during significant tissue remodeling [30].

Non-genomic vitamin D signaling may be especially pertinent during decidual transition, a phase in which stromal cells undergo significant structural and metabolic reconfiguration in a short timeframe. Rapid, non-genomic vitamin D signaling of this kind acting on mitochondrial function, cytoskeletal dynamics, and calcium-dependent pathways rather than through direct transcription has been described in other cell systems [31], and would plausibly support the swift adjustments in mitochondrial function, cytoskeletal dynamics, extracellular matrix interactions, and intracellular signaling that decidualizing stromal cells require to facilitate implantation and trophoblast invasion, although this has not been directly demonstrated in decidual cells specifically. Vitamin D can improve these activities by activating fast kinase pathways that increase cellular survival and mitigate stress-induced inflammatory damage. Consequently, impaired non-genomic regulation may result in less cellular plasticity in the decidua and heightened vulnerability to oxidative stress and inflammatory activation during early pregnancy [28].

#### 3.3.3. Genomic Non-Genomic Crosstalk and Epigenetic Regulation

Genomic and non-genomic mechanisms interact to synchronize immune control, metabolic adaptability, and decidual stability via vast the intracellular communication networks. The activation of PI3K/AKT signaling may affect downstream transcriptional regulators such as FOXO1, STAT proteins, mTOR-related pathways, and inflammatory transcription factors that play a role in decidualization and implantation. The interaction between these pathways enables vitamin D signaling to influence both immediate cellular responses and prolonged transcriptional regulation in the endometrium. The persistent dysregulation of these interrelated systems may lead to aberrant decidual adaptation and hindered maternal–fetal communication in recurrent pregnancy loss [19].

Furthermore, new molecular studies have shown that vitamin D transmission interacts with epigenetic regulatory pathways that govern the activity of endometrial cells. VDR-associated transcriptional complexes seem capable of recruiting chromatin-remodeling proteins, histone-modifying enzymes, and epigenetic cofactors that affect the accessibility of genes relevant to implantation [32]. Altered chromatin structure, aberrant methylation patterns, and dysregulated expression of non-coding RNAs associated with decidualization and immunological demonstration have been documented in recurrent reproductive failure. Given the broad range of biological processes influenced by vitamin D within the reproductive tract, a summary of its principal molecular and cellular actions at the maternal–fetal interface is provided in Table 1.

## 4. Decidualization, Progesterone Signaling, and Trophoblast Invasion

### 4.1. Cyclic Endometrial Changes and the Implantation Window

The endometrium undergoes continuous cyclical remodeling during the menstrual cycle, influenced by the coordinated actions of ovarian hormones, local inflammatory mediators, growth factors, immunological signaling pathways, and metabolic regulators. The implantation window is a limited timeframe in which the endometrium attains optimal receptivity for embryo adhesion [49]. This phase features a synchronized expression of adhesion molecules, chemokines, cytokines, angiogenic mediators, extracellular matrix proteins, and implantation-associated transcriptional regulators. Throughout this interval, several molecules, including HOXA10, osteopontin, leukemia inhibitory factor, glycodelin, integrins, and VEGF, exhibit elevated expression and have a direct role in embryo adhesion and trophoblast invasion. Disruption in the timing, amplitude, or geographic distribution of these molecular events may hinder embryo–endometrial communication, hence impacting the stability of implantation from its inception [11].

Furthermore, the behavior of immune cells in the endometrium undergoes substantial alterations throughout the peri-implantation phase. Uterine natural killer cells, macrophages, dendritic cells, and regulatory T-cells progressively penetrate decidualizing tissues, contributing to tissue remodeling, angiogenesis, inflammatory control, and maternal immunological tolerance [50]. Hence, implantation relies not only on hormonal synchronization but also on the establishment of a highly tailored immune milieu that facilitates trophoblast invasion and mitigates excessive inflammatory activation. Insufficient immunological adaptation may lead to repeated implantation failure and early pregnancy loss despite the existence of morphologically normal embryos [51].

Vitamin D signaling seems to be intricately associated with cyclic endometrial remodeling and molecular pathways relevant to implantation. The expression of VDR and CYP27B1 is elevated throughout the secretory and peri-implantation stages, indicating enhanced local sensitivity to vitamin D during decidualization and embryo attachment. Vitamin D may influence progesterone responsiveness, cytokine signaling, stromal cell differentiation, angiogenesis, oxidative stress responses, and immune cell recruitment during the implantation window [23].

**Figure 1:**
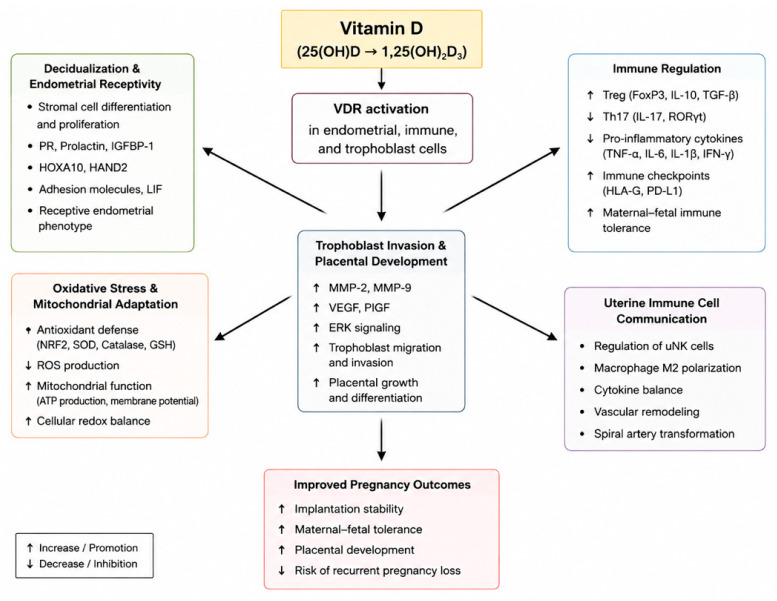
Integrated effects of vitamin D signaling on endometrial receptivity, immune tolerance, and implantation stability.

The inflammatory modulation during the implantation window is particularly susceptible to vitamin D-dependent signaling. This temporary pro-inflammatory milieu is essential for implantation, facilitating extracellular matrix remodeling and trophoblast invasion without harmful immune activation. Vitamin D signaling seems capable of regulating this equilibrium by inhibiting excessive NF-κB activation and modulating the generation of inflammatory cytokines [19]. Decreased vitamin D response may promote an endometrial milieu with ongoing inflammatory signaling, marked by elevated IL-17, TNF-α, and IFN-γ activity, eventually compromising implantation stability and maternal immunological tolerance. Recent transcriptomic and single-cell investigations have found several specialized stromal and immunological cell groups in the receptive endometrium that have diverse functional functions during implantation [52]. Certain decidual fractions have transcriptional patterns that facilitate implantation, enriched in progesterone-responsive, angiogenic, and immune-regulatory pathways, whereas others manifest inflammatory or stress-related phenotypes linked to reproductive failure. Alterations in the differentiation and temporal control of these cell types may significantly contribute to recurrent miscarriage and implantation issues [53]. Vitamin D signaling may play a role in sustaining this intricate physiological equilibrium by coordinating the control of inflammatory, metabolic, and transcriptional networks throughout the receptive phase of the menstrual cycle (Figure 1).

Figure 1 shows a schematic diagram of the major molecular and cellular pathways regulated by vitamin D at the maternal–fetal interface. Vitamin D binds to the vitamin D receptor (VDR) in endometrial, immune, decidual and trophoblast cells [4,9], where it is activated to 1,25(OH)2D3 and promotes decidualization [4], immune regulation [19], adaptation to oxidative stress, trophoblast invasion, mitochondrial homeostasis, and placental development [10]. Dysregulation of these interlinked pathways may underlie implantation instability and recurrent pregnancy loss. The oxidative stress, trophoblast invasion, and mitochondrial components of this schematic are elaborated with dedicated evidence in Section 4, Section 5 and Section 6. Not all pathways shown carry equivalent evidentiary support: decidualization, immune regulation, and trophoblast invasion are supported by both experimental and clinical human data, whereas the mitochondrial and oxidative stress connections are derived predominantly from experimental and preclinical models.

### 4.2. Physiological Mechanisms of Decidualization

Decidualization is a complicated differentiation process in human reproductive biology that transforms the endometrium from a cyclically hormonally sensitive tissue into a specialized maternal structure capable of sustaining implantation and placental development. Progesterone rises post-ovulation, prompting significant biochemical, morphological, vascular, immunological, and metabolic changes of the endometrial stroma [54]. Fibroblast-like stromal cells gradually transform their initial shape and adopt a secretory decidual phenotype, marked by increased cell volume, modified cytoskeletal architecture, elevated biosynthetic activity, and significant transcriptional reprogramming. This transition establishes a transient maternal milieu that facilitates embryo attachment, regulates trophoblast invasion, and maintains tissue homeostasis throughout early pregnancy [55].

The decidual process transcends a mere reaction to progesterone and is defined by the coordinated activation of many communication within cell networks. The differentiation of stromal cells is regulated by the interplay of cyclic AMP pathways, progesterone receptor pathways, inflammatory mediators, growth factors, metabolic sensors, and chromatin remodeling processes [33]. As stromal cells acquire decidual traits, there is a gradual rise in the expression of prolactin, insulin-like growth factor binding protein-1, FOXO1, HAND2, HOXA10, and many progesterone-regulated genes. These molecular alterations are reflected in substantial modifications in the extracellular matrix composition, intercellular communication, angiogenic interaction, and secretory functions. Impaired coordination of these transcriptional processes may destabilize implantation before affecting embryonic development, indicating that recurrent pregnancy loss may stem from inadequate maternal adaptation rather than exclusively from embryonic defects [56].

Decidual maturation entails a significant reconfiguration of cellular energetics, aligning with the elevated metabolic requirements of implantation and early placentation. Tissue remodeling and the synthesis of mediators related to implantation need stromal cells undergoing decidual differentiation for improving mitochondrial respiration, glucose absorption, amino acid turnover, lipid morphology, and synchronized oxidative phosphorylation [57]. Mitochondria are essential regulators of this process for ATP synthesis, as well as the control of calcium signaling, reactive oxygen species generation, apoptotic vulnerability, and intracellular stress responses. Thus, impaired mitochondrial adaptation may jeopardize the survival of stromal cells and the decidua’s ability to support trophoblast invasion and embryo implantation. Impaired mitochondrial function is increasingly associated with aberrant decidualization, unstable implantation, and recurrent reproductive failure [33].

Vitamin D signaling seems to be increasingly interconnected with the pathways of decidual metabolism and differentiation. In the secretory phase, endometrial stromal cells have the vitamin D receptor and CYP27B1, facilitating the local activation of vitamin D during decidual transformation [12]. The activation of vitamin D transmission affects genes related to stromal cell maturation, inflammatory control, oxidative defense, extracellular matrix organization, and cell adhesion. In addition to transcriptional regulation, vitamin D seems to influence mitochondrial homeostasis, intracellular calcium equilibrium, and stress-response pathways in decidual cells. These effects may be especially significant after implantation, when stromal cells encounter rapid changes in metabolic and inflammatory conditions [58].

The inflammatory signaling of decidualization must be carefully managed. The implantation process necessitates a temporary pro-inflammatory milieu conducive to tissue remodeling, vascular permeability, and trophoblast contact. However, persistent or severe inflammatory activation might destabilize decidual tissues and induce cellular damage [33]. Chronic inflammatory stimuli result in impaired differentiation, modified cytokine production, heightened oxidative stress, mitochondrial dysfunction, and increased vulnerability to apoptosis in stromal cells. The activation of inflammatory pathways associated with NF-κB seems to be especially relevant. Vitamin D signaling may mitigate these effects by inhibiting excessive inflammatory activation and promoting tolerogenic cytokine profiles in the decidual microenvironment [28].

Oxidative stress has become a significant factor influencing decidual competence and the durability of implantation. Physiological concentrations of reactive oxygen species in intracellular signaling and tissue remodeling increase following implantation [59]. Excessive buildup of ROS might impair the mitochondrial membrane, lipids, proteins, and DNA in decidual tissues, thereby hindering stromal cell differentiation and fostering inflammatory activation. Prolonged oxidative damage to cells might result in senescence or death, ultimately compromising the structural and functional integrity of the implantation environment. Vitamin D is likely tightly linked to antioxidant defense mechanisms, including NRF2 activation, glutathione metabolism, superoxide dismutase activity, and modulation of intracellular redox homeostasis, which may provide protection against oxidative decidual damage [60].

There is a growing interest in the function of cellular senescence in implantation biology. Regulated senescence seems essential for physiological decidual remodeling and trophoblast direction, facilitating tissue turnover and local inflammatory signaling necessary for implantation [61]. Excessive or premature senescence may result in a persistent pro-inflammatory decidual phenotype characterized by the secretion of matrix-degrading enzymes, inflammatory cytokines, and stress-related mediators, together referred to as the senescence-associated secretory phenotype. Such alterations may disrupt implantation and adversely impact placental development. Modifications in vitamin D transmission have been linked to mechanisms that govern cellular ageing, mitochondrial stress responses, autophagy, and inflammatory senescence. This indicates that a deficiency in vitamin D response may play a role in premature decidual ageing in women experiencing recurrent pregnancy loss [62].

### 4.3. Vitamin D Regulation of Decidual Stromal Cells

Decidual stromal cells are essential components of the implantation microenvironment, functioning as structural elements of the endometrium and as active regulators of immune adaptation, trophoblast invasion, vascular remodeling, and early placental development [63]. In the secretory phase, progesterone-induced differentiation transforms endometrial stromal fibroblasts into specialized decidual cells capable of producing cytokines, growth factors, extracellular matrix proteins, metabolic mediators, and implantation-related signaling molecules. This differentiation process requires precise coordination among endocrine communication, transcriptional regulation, intracellular metabolism, and inflammatory management. Impairment of stromal-cell maturation has been increasingly associated with implantation failure, recurrent miscarriage, and placental malfunction [64].

Vitamin D transmission seems to be closely implicated in many phases of decidual stromal cell development. Throughout decidualization, elevated expression of both the vitamin D receptor and CYP27B1 indicates enhanced local sensitivity to vitamin D throughout the implantation period. Vitamin D modulates the expression of genes associated with stromal maturation, cellular adhesion, immunological control, oxidative balance, and progesterone responsiveness via the activation of VDR-dependent transcriptional pathways. Experimental investigations have shown that vitamin D signaling modulates the production of prolactin and IGFBP-1, two established markers of decidual differentiation, indicating a direct role in the control of stromal cell phenotypic acquisition [65].

A primary purpose of decidual stromal cells is to provide a regulated inflammatory milieu that facilitates embryo implantation while preventing excessive immune activation. Decidual cells regulate the recruitment and functional activity of uterine natural killer cells, macrophages, dendritic cells, and regulatory T-cells by secreting cytokines and chemokines during early implantation [66]. Vitamin D seems to affect several pathways of communication by diminishing pro-inflammatory mediators and promoting tolerogenic immunological responses. Hence, diminished vitamin D response in stromal cells may facilitate prolonged inflammatory activation during implantation and disrupt maternal immunological adaption to pregnancy [19].

Decidualization involves significant metabolic adaptation, characterized by improved mitochondrial activity, synchronized oxidative phosphorylation, amino acid turnover, glucose utilization, and lipid remodeling. Vitamin D seems to modulate mitochondrial stability and intracellular energy balance via pathways associated with PI3K/AKT indicating, calcium, oxidative stress, and mitochondrial membrane integrity. Consequently, impaired vitamin D regulation may limit stromal cells’ capacity to provide the energy requirements for implantation and trophoblast maintenance [67].

The modulation of oxidative stress in decidual stromal cells is significantly influenced by vitamin D activity. Physiological decidualization entails the regulated generation of reactive oxygen species, which is essential for communication within cells and tissue remodeling. Excessive buildup of ROS may hinder stroma-cell differentiation, stimulate the production of inflammatory cytokines, destabilize mitochondrial function, and activate apoptotic pathways. Vitamin D transmission seems to stimulate the antioxidant defense system via NRF2 activation, modulation of glutathione metabolism, and control of antioxidant enzymes, including superoxide dismutase and catalase. Thus, impaired oxidative adaption in decidual stromal cells may result in implantation instability and recurrent pregnancy loss [68].

The interplay between vitamin D transmission and progesterone responsiveness has garnered more interest recently. Progesterone is the principal hormonal initiator of decidualization; nevertheless, effective stromal cell differentiation requires further transcriptional and metabolic adjustments after progesterone receptor activation. Vitamin D seems to interact with many progesterone-responsive mechanisms that regulate decidual development, cellular survival, and inflammatory balance. Decreased VDR signaling may impair stromal cell sensitivity to progesterone, despite normal circulating levels of the hormone, thereby contributing to the functional progesterone resistance seen in women with recurrent reproductive failure [7,12].

Vitamin D seems to play a role in the modulation of extracellular matrix dynamics inside the decidual compartment. Decidual stromal cells actively modulate collagen organization, integrin expression, matrix metalloproteinase activity, and tissue elasticity following implantation. These structural routes affect trophoblast migration and embryo attachment, while preserving decidual integrity. Modified vitamin D transmission may disrupt communication between stromal cells and invading trophoblasts, as well as affect the reshaping of the extracellular matrix, eventually leading to compromised placental anchoring and vascular adaption in early pregnancy.

### 4.4. Progesterone Resistance and Defective Decidual Transformation

Much of the molecular detail in this section is derived from human decidual stromal cell cultures and endometrial biopsy studies; the specific transcriptional and inflammatory mechanisms described have not been established through in vivo human intervention studies, and the interaction with vitamin D signaling in particular rests mainly on in vitro and small observational cohorts rather than confirmatory clinical data.

Progesterone is the primary hormonal regulator of decidualization, converting the proliferative endometrium into a differentiated decidual tissue capable of supporting implantation. Progesterone resistance, a diminished cellular response to progesterone despite normal circulating hormone levels, has drawn particular interest in women with recurrent implantation failure and recurrent pregnancy loss, and may arise from aberrant progesterone receptor expression or signaling, inflammatory interference, epigenetic dysregulation, or metabolic instability in stromal cells [69,70].

Decidual stromal cells with compromised progesterone signaling show reduced expression of prolactin, IGFBP-1, FOXO1, HOXA10, and implantation-related adhesion proteins [71]. Notably, histological maturation of the endometrium may appear intact even when these underlying molecular abnormalities are present, meaning that standard diagnostic assessment can miss this dysfunction entirely [72].

Inflammatory and metabolic disruption converge on the same downstream pathway to impair progesterone response. Chronic NF-κB activation and elevated IL-6, TNF-α, IL-17, and IFN-γ disrupt progesterone receptor functionality and downstream transcription [40]. This is compounded by mitochondrial dysfunction: decidualizing stromal cells require substantial metabolic flexibility, and oxidative stress, reduced ATP synthesis, altered calcium homeostasis, and mitochondrial membrane instability during this transition can disrupt progesterone-mediated transcriptional programs and stromal cell viability through the same inflammatory and oxidative mechanisms [73].

Vitamin D signaling intersects with progesterone-dependent decidual pathways at multiple points in this cascade. VDR activation influences the expression of progesterone-responsive genes governing stromal differentiation, inflammatory control, and angiogenesis, and experimental evidence suggests that vitamin D may improve decidual-cell responsiveness to progesterone partly by downregulating the inflammatory pathways described above [19,74]. Taken together, current evidence supports progesterone resistance as a multifactorial disorder combining endocrine, inflammatory, oxidative, and transcriptional dysfunction, rather than a simple hormone-concentration abnormality, a pattern consistent with the finding that many women with recurrent pregnancy loss have normal serum progesterone despite underlying decidual signaling defects [75]. Progesterone resistance also affects the interaction between decidual stromal cells and invading trophoblasts. Decidual cells guide trophoblast migration through cytokines, chemokines, adhesion molecules, and angiogenic mediators; inadequate decidual maturation may disrupt this crosstalk and the balance between controlled trophoblast invasion and tissue integrity [76].

### 4.5. Vitamin D and Trophoblast Invasion

The mechanisms described in this section are derived primarily from trophoblast cell-line and explant culture models, with a smaller contribution from animal studies of spiral artery remodeling; direct human in vivo evidence linking vitamin D signaling to trophoblast invasion is limited, and the clinical relevance described below should be read as biologically plausible rather than established in humans.

Successful implantation requires not only sufficient decidual transformation, but also the capacity of trophoblast cells to establish a controlled interaction with tissues of the mother during the earliest stages of placentation. After implantation of the embryo, trophoblasts progressively invade the decidua and remodel maternal spiral arteries to ensure oxygen and nutrient supply to the developing conceptus. This process necessitates a highly coordinated communication network between trophoblastic tissue, decidual stromal cells, endothelial structures, immune-cell populations, extracellular matrix components, and local metabolic pathways. Both insufficient and excessive trophoblast invasion may compromise placental development and pregnancy stability, indicating the importance of precise regulatory control during early gestation.

Trophoblast invasion is characterized by dynamic regulation of cellular adhesion, motility, proteolytic activity, angiogenesis and immune adaptation. Extravillous trophoblasts acquire an invasive phenotype that penetrates the decidual extracellular matrix and transforms spiral arteries into low-resistance vessels suitable for placental perfusion. This tightly orchestrated process involves matrix metalloproteinases, integrins, cadherins, chemokines and growth factors. Aberrant regulation of these pathways may impair placental anchoring, limit vascular remodeling, and increase the risk of early pregnancy loss or placental dysfunction later in gestation [77].

Vitamin D signaling seems to be increasingly involved in modulation of trophoblastic behavior during implantation and placentation. Vitamin D activity is locally regulated within the maternal–fetal interface as both trophoblasts and decidual tissues express VDR and CYP27B1. Activation of vitamin D pathways affects genes related to trophoblast differentiation, migration, invasion, angiogenesis, and inflammatory regulation [78]. Experimental data suggest that vitamin D may stimulate controlled trophoblast invasion through regulation of matrix metalloproteinases, VEGF signaling, adhesion molecules and extracellular matrix interaction pathways.

Regulation of matrix remodeling is one of the most important mechanisms through which vitamin D may influence trophoblast invasion. Controlled degradation of the decidual extracellular matrix is required to allow trophoblast penetration while maintaining tissue integrity and preventing excessive invasion. Matrix metalloproteinases such as MMP-2 and MMP-9 play crucial roles in this process by degrading collagen and basement membrane components surrounding maternal vessels and stromal tissues. Vitamin D signaling seems capable of modulating expression and activity of these proteolytic enzymes along with their endogenous inhibitors, thus affecting the balance between tissue remodeling and structural stability during placentation [79].

Angiogenesis and vascular adaptation in early pregnancy also seem to be in close association with vitamin D activity. Trophoblasts, endothelial cells, decidual stromal cells, macrophages, and uterine natural killer cells interact in a coordinated fashion to drive spiral artery remodeling. Vitamin D has been linked to regulation of VEGF, angiopoietins, endothelial nitric oxide synthesis and vascular inflammatory signaling. Impaired vitamin D responsiveness may thus interfere with vascular transformation in the decidua and contribute to abnormal placental perfusion in early gestation. Such disturbances may first be reflected as instability of implantation and later predispose to complications related to placental insufficiency [62,80].

Equally important for successful placentation is the immunological environment surrounding invading trophoblasts. Trophoblast cells come into immediate contact with maternal immune cells after implantation, and must induce local tolerance while preventing excessive inflammatory activation. Vitamin D signaling appears to be able to modulate this milieu through effects on cytokine production, antigen presentation, macrophage polarization, and regulatory T-cell expansion. Reduced responsiveness to vitamin D could promote excessive inflammatory signaling in decidual tissues and conditions that are detrimental to trophoblast survival and migration [81].

Interactions between trophoblasts and uterine natural killer cells are another key component of implantation biology influenced by vitamin D signaling. Unlike peripheral NK cells, decidual NK populations mainly facilitate trophoblast invasion and vascular remodeling by secreting angiogenic mediators and growth factors. Abnormal activation or altered cytokine production in these cell populations may disrupt trophoblast guidance and contribute to implantation failure or recurrent miscarriage. Vitamin D seems able to modulate decidual NK-cell phenotype and inflammatory activity, and thus could influence the balance between trophoblast support and cytotoxic immune activation.

A critical evaluation of this evidence is warranted. The link between vitamin D and MMP-2/MMP-9 activity rests on a small number of in vitro trophoblast and endometrial cell studies showing VDR-dependent changes in proteolytic enzyme expression; no human study has directly demonstrated that vitamin D status determines MMP activity or invasion depth in vivo. The vitamin D–VEGF relationship is similarly supported mainly by cell culture and animal placental models, with human data limited to correlational associations between circulating vitamin D and angiogenic markers rather than functional proof of a causal link. Evidence connecting vitamin D to integrin expression in trophoblasts is the weakest of the three, derived almost entirely from in vitro adhesion assays. Given this evidence base, the clinical relevance of vitamin D-modulated trophoblast invasion to recurrent pregnancy loss in humans remains speculative, and should be understood as a plausible mechanistic hypothesis rather than a demonstrated contributor to disease.

### 4.6. Molecular Pathways Linking Decidual Dysfunction to Recurrent Pregnancy Loss

A significant genetic abnormality linked to recurrent pregnancy loss is disrupted stromal cell differentiation. Under physiological settings, decidual transition requires the activation of precisely coordinated transcriptional programs that regulate cellular maturation, secretory function, angiogenesis, and inflammatory regulation. The dysregulated pathways in women experiencing recurrent miscarriage entail altered expression of prolactin, IGFBP-1, FOXO1, HOXA10, HAND2, and progesterone-responsive genes that are critical for implantation support. The inadequate activation of these transcriptional networks may lead to stromal cells remaining in a semi-differentiated state, rendering them incapable of facilitating embryo implantation and trophoblast invasion [11].

A further significant mechanism linking decidual dysfunction to reproductive failure is the impairment of progesterone-dependent signaling. Circulating progesterone levels may be normal; however, decidual stromal cells may have diminished reactivity at the receptor or post-receptor level [82]. Such anomalies may undermine the subsequent transcriptional activity required for endometrial development and implantation stability. Decreased reactivity to progesterone has been associated with ongoing inflammatory indicating, irregular extracellular matrix remodeling, atypical cytokine production, and impaired vascular restructuring in the decidual [83].

Inflammatory dysregulation has been recognized as a critical biological feature of aberrant decidualization. Implantation requires a regulated inflammatory milieu that facilitates tissue remodeling and trophoblast invasion while preventing harmful immune activation. Recurrent pregnancy loss is marked by elevated levels of pro-inflammatory cytokines, including IL-6, IL-17, TNF-α, and IFN-γ in decidual tissues, and impaired anti-inflammatory signaling mediated by IL-10 and TGF-β. Hyperinflammatory activation may hinder trophoblast migration, exacerbate cellular stress responses, impair vascular remodeling, and undermine maternal immunological tolerance to the embryo [3,84].

Regulatory mechanisms associated with NF-κB seem to be relevant in this situation. Extended NF-κB activation in decidual stromal cells promotes the production of inflammatory mediators, chemokines, adhesion molecules, and stress-response proteins, potentially worsening local tissue damage. Chronic inflammatory activation may potentially affect progesterone responsiveness and the development of decidual cells. Vitamin D signaling may regulate excessive NF-κB signaling under physiological settings, suggesting that impaired vitamin D response might contribute to prolonged inflammatory activity in the implantation environment [85].

Aberrant immune-cell interactions within decidual tissues also exacerbate implantation instability. Decidual stromal cells often modulate interactions with uterine natural killer cells, macrophages, dendritic cells, and regulatory T-cells via the release of cytokines, chemokines, extracellular vesicles, and metabolic mediators. This network of contacts is crucial for sustaining maternal immunological tolerance and the potential for tissue remodeling. Disruptions in immunological communication in the decidua with recurrent pregnancy loss may lead to heightened cytotoxicity, atypical macrophage polarization, impaired Treg expansion, and elevated Th17 inflammatory activity [86].

A significant molecular mechanism between impaired decidualization and recurrent miscarriage is oxidative stress. Excessive buildup of ROS may damage mitochondrial membranes, DNA, proteins, and lipids in decidual tissues. Stromal cells subjected to persistent oxidative stress demonstrate diminished differentiation capacity, heightened vulnerability to apoptosis, and augmented inflammatory activation. Oxidative damage may hinder trophoblast invasion and vascular adaption during early placentation, hence destabilizing pregnancy development [87].

Mitochondrial dysfunction seems to be closely associated with oxidative decidual damage. Decidual transformation is linked to significant metabolic adaptability and heightened mitochondrial activity to facilitate cellular differentiation and secretory functions related to implantation. Decreased ATP synthesis, unstable mitochondrial membranes, disrupted calcium homeostasis, and compromised oxidative phosphorylation may adversely affect decidual cell survival and implantation capability. Mitochondria also modulate inflammatory activation and apoptotic pathways in stromal cells, linking metabolic failure directly to tissue stability at the maternal–fetal interface [56].

Ferroptosis and other controlled cell death processes have recently been associated with recurrent reproductive failure. Ferroptosis is a kind of cell death characterized by iron-dependent lipid peroxidation and oxidative membrane degradation, which may adversely affect the functionality of decidual and placental cells [88]. Epigenetic dysregulation is another significant factor in aberrant decidual transformation. Altered DNA methylation patterns, histone modifications, chromatin remodeling, and non-coding RNA expression have been documented in decidual tissues of women experiencing recurrent pregnancy loss. Numerous epigenetic anomalies pertain to mechanisms regulating stromal differentiation, inflammatory modulation, oxidative stress responses, and gene expression associated with implantation. The results indicate that women experiencing recurrent miscarriage have enduring changes in endometrial cell programming that extend beyond isolated hormonal irregularities in particular cycles [73].

Aberrant remodeling of the extracellular matrix exacerbates implantation failure and early pregnancy instability. Decidual stromal cells modulate collagen structure, integrin expression, matrix metalloproteinase activity, and tissue elasticity following implantation. Disruptions in these structural processes may hinder embryo attachment, trophoblast migration, and vascular restructuring. Excessive upgrading of the matrix may destabilize implantation sites, while inadequate remodeling may restrict trophoblast invasion and placental attachment. The molecular mechanisms discussed throughout this section converge toward a common model of implantation instability characterized by simultaneous disturbances in decidual, inflammatory, oxidative, and metabolic pathways. These interactions are summarized in Table 2.

## 5. Maternal–Fetal Immune Escape and Immune Dysregulation

### 5.1. Maternal Immune Tolerance During Early Pregnancy

Pregnancy is a novel immunological condition when the maternal immune system must concurrently defend against infections and accommodate a genetically disparate embryo. The growing conceptus exhibits paternal antigens, so resembles a semi-allogeneic graft. Successful implantation and placental development often transpire without immune rejection, indicating the presence of highly specialized regulatory systems at the maternal–fetal interface. The failure of these adaptive mechanisms may significantly contribute to recurrent pregnancy loss, implantation failure, and aberrant placentation [94].

Robust immune activity in the endometrium correlates with early implantation. Decidual tissues are abundant in uterine natural killer cells, macrophages, dendritic cells, regulatory T-cells, and specialized stromal cell populations, which may coordinate local immunological adaption. These cellular interactions provide a managed inflammatory milieu essential for tissue remodeling, trophoblast invasion, angiogenesis, and vascular transformation. Thus, physiological implantation involves not only immune suppression, but the creation of a calibrated equilibrium between inflammatory activity and immunological tolerance [95].

Decidual stromal cells are essential in this process and maintain continuous interaction with the local immune populations. In addition to their structural functions, decidual cells also participate in cytokine production, chemokine gradients, extracellular matrix structure, metabolic indicating, and the recruitment of tolerogenic immune cell subsets. These interactions influence the development and function of uterine natural killer cells, macrophages, dendritic cells, and regulatory T-cells at the implantation site. Alterations in decidual–immune interactions may undermine maternal tolerance and establish an inflammatory milieu detrimental to normal placental development [96].

The cytokine environment in the decidua experiences dynamic alterations during implantation and early pregnancy. Initially, inflammatory mediators such as IL-6, TNF-α, and chemokines must be generated in a regulated manner to facilitate tissue remodeling and trophoblast invasion. During implantation, anti-inflammatory and tolerogenic mechanisms progressively dominate to suppress excessive immune activation against the trophoblastic tissue. The preservation of this delayed immunological shift seems essential for implantation stability. If inflammatory signaling persists beyond the normal implantation period, placental development may be impaired, hence increasing the risk of miscarriage [97].

Regulatory T-cells are a crucial immunological population implicated in maternal tolerance. The expansion of Treg populations during early gestation suppresses excessive inflammatory responses and facilitates the immunological tolerance of fetal antigens. Reduced Treg activity has been linked to recurrent miscarriage and implantation failure. Numerous studies indicate that insufficient growth or functional instability of Treg populations may allow for ongoing Th1- and Th17-mediated inflammatory activity in decidual tissues. Such anomalies may disrupt trophoblast invasion, vascular adaption, and placental development in the first stages of pregnancy [98].

Uterine natural killer cells possess highly specific roles distinct from those of peripheral NK populations. Decidual NK cells primarily facilitate trophoblast invasion and vascular restructuring via the secretion of angiogenic mediators, cytokines, and growth factors, rather than mostly functioning as cytotoxic effectors. Abnormal activation or modified phenotype of these cells may lead to recurrent reproductive failure due to excessive cytotoxicity, inflammatory activation, or defective remodeling of spiral arteries. Consequently, appropriate modulation of decidual NK-cell activity is crucial for sustaining implantation stability and placental development [99].

Decidual tissues include macrophages that facilitate immunological adaption during pregnancy. Physiological implantation is marked by the prevalence of alternatively activated M2-like macrophages, which possess tissue-remodeling, angiogenic, and anti-inflammatory capabilities [100]. The transition to pro-inflammatory M1 phenotypes may result in excessive cytokine production, oxidative stress, extracellular matrix damage, and trophoblast malfunction. Changes in macrophage polarization have been seen in women experiencing recurrent pregnancy loss, supporting the concept that dysregulation of the innate immune system contributes to implantation instability [101].

Dendritic cells contribute to maternal immunological tolerance by facilitating antigen presentation and modulating T-cell development. Tolerogenic dendritic cell morphologies in decidual organs promote Treg growth and inhibit excessive inflammatory responses. Abnormal dendritic-cell activation may lead to altered local immunological balance and heightened maternal immune recognition of trophoblastic antigens [76].

Numerous immune-regulatory mechanisms seem to be closely associated with vitamin D signaling. Various immune cell types implicated in implantation express the vitamin D receptor, including Treg cells, macrophages, dendritic cells, and uterine natural killer cells. Activation of vitamin D transmission influences cellular differentiation, cytokine synthesis, and both inflammatory and tolerogenic immune responses in decidual tissues [28]. The immunomodulatory effects of vitamin D at the maternal–fetal interface may be elucidated via many methods. Vitamin D suppresses NF-κB-mediated inflammatory activation and promotes the synthesis of anti-inflammatory cytokines, including IL-10 and TGF-β. Simultaneously, vitamin D seems to modulate the inflammatory response of Th17 cells and enhance the proliferation of regulatory T-cell populations [102].

### 5.2. Regulatory T-Cells and Th17 Imbalance

The imbalance between regulatory T-cells and T-helper 17 cells has become one of the most thoroughly investigated processes linked to immunological disorders in recurrent pregnancy loss. Physiological implantation requires a regulated inflammatory response during the first phases of embryo attachment, followed by the gradual formation of a tolerogenic immunological milieu conducive to placental growth [103]. Regulatory T-cells play a crucial role in this shift by inhibiting excessive immune activation and promoting maternal immunological tolerance to fetal antigens. Conversely, Th17 cells mostly function as mediators of inflammatory reactions, facilitating the recruitment and activation of immunological pathways linked to tissue damage and chronic inflammation. Maintaining a balance between these two groups is crucial for successful pregnancy [104].

Regulatory T-cells aggregate in the decidual tissues throughout the peri-implantation phase and early gestation. These cells suppress inflammatory responses by secreting IL-10, TGF-β, and other immunoregulatory mediators, while also restraining the excessive activation of effector T-cells, natural killer cells, and antigen-presenting cells [34]. Treg populations also play a role in vascular remodeling, trophoblast tolerance, and the maintenance of decidual homeostasis. Decreased quantities or compromised functional activity of Treg cells have consistently been seen in women experiencing recurrent miscarriage, suggesting a deficiency in maternal immunological adaption during implantation and placentation [105].

Th17 cells have almost contradictory physiologic effects at the maternal–fetal contact. These cells produce pro-inflammatory cytokines, including IL-17, IL-21, and IL-22, which attract neutrophils, increase inflammation, increase oxidative stress, and activate tissue-destructive mechanisms. Restricted Th17 activity may contribute to physiological implantation-related inflammation, but prolonged or excessive Th17 responses seem to be harmful to pregnancy maintenance. Recurrent pregnancy loss, implantation failure, and aberrant decidual inflammatory activation have been repeatedly linked to heightened Th17-cell populations and higher IL-17 levels [36].

The link between Treg and Th17 differentiation is notably intertwined, as both lineages originate from naïve CD4+ T-cells in response to local cytokine and metabolic circumstances. TGF-β alone often encourages Treg differentiation, but its combination with IL-6, IL-1β, or IL-23 facilitates Th17 polarization. Decidual inflammatory circumstances significantly influence the equilibrium between tolerogenic and inflammatory T-cell responses. Chronic inflammatory activation during implantation may alter this equilibrium in favor of Th17 dominance and impair maternal immunological tolerance in early pregnancy [36].

Vitamin D signaling seems to be closely associated with the modulation of this immunological equilibrium. The activation of the vitamin D receptor affects the differentiation, stability, and functional activity of Treg and Th17 populations via several transcriptional and cytokine-mediated pathways [106]. Vitamin D promotes the proliferation of FoxP3-positive regulatory T-cells while concurrently inhibiting pathways associated with Th17 differentiation and IL-17 synthesis. Hence, diminished vitamin D response promotes the establishment of a pro-inflammatory decidual milieu, marked by heightened Th17-mediated activity and insufficient immunological tolerance [107].

Multiple molecular mechanisms may explain the impact of vitamin D on T-cell polarization. VDR activation inhibits inflammatory transcription factors such STAT3 and RORγt, which play essential roles in Th17 development. Vitamin D seems to promote FoxP3 expression while concurrently stabilizing the regulatory T-cell phenotype. Such pathways may directly influence the capacity of decidual organs to sustain immunological homeostasis following implantation [108]. A significant route via which vitamin D may affect Treg/Th17 dynamics is cytokine modulation. Vitamin D suppresses the synthesis of IL-6, IL-1β, TNF-α, and IL-23, cytokines closely linked to Th17 proliferation and the enhancement of inflammation. Emerging research indicates that the volatility of Tregs, rather than their absolute lack, may lead to recurrent miscarriage. In inflammatory and oxidative environments, regulatory T-cells may forfeit their suppressive abilities and adopt traits akin to those of inflammatory effector cells. This phenotypic instability may undermine maternal immune tolerance, even with seemingly adequate Treg populations. Vitamin D signaling is crucial for the preservation of Treg stability by modulating transcriptional and epigenetic pathways that govern FoxP3 expression and suppressive activity [35].

Furthermore, an aberrant Treg/Th17 equilibrium may directly affect trophoblast behavior. IL-17-mediated inflammatory activation has been associated with compromised trophoblast migration, heightened oxidative damage, endothelial dysfunction, and aberrant vascular remodeling. Conversely, a tolerogenic immunological milieu facilitates trophoblast invasion and placentation via the release of growth factors and anti-inflammatory mediators [37].

Recurrent pregnancy loss is widely seen as a condition of impaired immunological adaptation, namely, the inability to transition from implantation-related inflammation to sustained maternal tolerance. An imbalance between regulatory T-cells and Th17 populations seems to be a significant immunological hallmark of this process. Vitamin D signaling interacts with many pathways that govern T-cell differentiation, cytokine synthesis, oxidative stress responses, metabolic adaptability, and transcriptional control in decidual tissues. The disruption of these interrelated systems may account for a significant fraction. Immune disturbances represent one of the most consistently reported findings in recurrent pregnancy loss [109]. The major alterations described in the literature and their potential relationship with vitamin D signaling are summarized in Table 3.

### 5.3. Uterine Natural Killer Cells in Implantation and Pregnancy Maintenance

Uterine natural killer cells are the predominant immune cell population in the decidua during early pregnancy, playing a crucial role in implantation and placental development. Unlike peripheral natural killer cells, which largely serve as cytotoxic effectors in antiviral and anticancer immunology, decidual NK cells have a specialized phenotype exclusive to reproductive physiology. Instead of inducing tissue damage, these cells modulate trophoblast invasion, vascular remodeling, angiogenesis, extracellular matrix organization, and local immune response during the first phases of gestation. Their activity must be carefully regulated, as both insufficient and excessive NK-cell activation may jeopardize implantation stability [111].

In the secretory phase, decidual NK cells amass in the endometrium due to the effects of progesterone, cytokines, chemokines, and local decidual signaling pathways. Their population increases throughout the peri-implantation phase, ultimately constituting up to 70% of the leukocytes in first-trimester decidual tissue. This significant enhancement highlights their biological relevance in implantation. Decidual NK cells have a unique phenotype compared to circulating NK populations, characterized by less cytotoxicity and elevated production of angiogenic mediators, growth factors, cytokines, and tissue-remodeling molecules essential for placental development [38].

The primary role of uterine NK cells is to modulate trophoblast invasion and transformation of spiral arteries. During implantation, extravillous trophoblasts infiltrate maternal decidual tissues and progressively remodel spiral arteries into low-resistance channels capable of sustaining placental perfusion. Decidual NK cells release VEGF, placental growth factor, angiopoietins, and matrix-remodeling enzymes that assist in this process by directing trophoblast movement and vascular adaption. Disruptions in NK-cell signaling may affect placental anchoring, resulting in implantation failure or aberrant placental development [39].

The connection between decidual NK cells and trophoblasts relies on a complicated network of receptor–ligand interactions. Trophoblast cells carry non-classical HLA molecules, including HLA-C, HLA-E, and HLA-G, which interact with inhibitory and activating receptors present on NK cells. These interactions regulate trophoblast tolerance, vascular remodeling, cytokine synthesis, and immune cell activation in decidual tissues. Aberrant receptor activation may induce a change in NK-cell behavior towards heightened inflammatory activation or cytotoxicity, perhaps leading to recurrent pregnancy loss [47].

Decidual NK cells actively participate in regulating the local inflammatory equilibrium following implantation. Physiological implantation requires temporary inflammation sufficient for rebuilding of tissues and trophoblast invasion while preventing uncontrolled immune-mediated tissue damage. NK cells facilitate this equilibrium by releasing cytokines such as interferon-gamma (IFN-γ), interleukin-8 (IL-8), granulocyte-macrophage colony-stimulating factor (GM-CSF), and angiogenic mediators that coordinate vascular adaptation and decidual communication. An overproduction of inflammatory cytokines or a dysregulated activation of cytotoxic pathways may result in destabilization of the maternal–fetal interface and hinder placental development [112].

Numerous investigations have shown modified NK-cell activity in women experiencing recurrent miscarriage; however, the precise pathogenic significance of these changes remains unclear. Recurrent reproductive failure has been associated with increased cytotoxicity, modified receptor expression, intensified inflammatory communication, and atypical distribution of decidual NK cells [113]. In some women, there is heightened peripheral NK-cell activation, while in others, the abnormalities mostly occur in the decidual tissues. The results suggest that recurrent pregnancy loss may result from dysregulated NK-cell activity rather than only from changes in NK-cell levels.

The local decidual microenvironment significantly influences the phenotypic and activity of NK cells. Decidual stromal cells modulate NK-cell recruitment, differentiation, and cytokine synthesis by the release of IL-15, TGF-β, chemokines, and extracellular vesicles. Macrophages, dendritic cells, trophoblasts, and regulatory T-cells further support the specialized decidual NK-cell phenotype. The disruption of this communication network may influence NK-cell behavior and result in inflammatory instability at implantation sites [38,76].

Vitamin D signaling is increasingly associated with the modulation of decidual NK-cell function. NK cells possess functional vitamin D receptors, and the activation of vitamin D receptor-dependent pathways influences cytokine synthesis, inflammatory responses, cellular maturation, and cytotoxic activity [114]. Vitamin D seems capable of suppressing excessive inflammatory activation and fostering a more tolerogenic NK-cell phenotype in decidual tissues. Various molecular processes may be implicated in the interaction between vitamin D transmission and NK-cell control. Vitamin D diminishes the synthesis of cytokines linked to cytotoxic activation and inhibits NF-κB-associated inflammatory pathways. Simultaneously, vitamin D may augment the production of tolerogenic mediators and regulate receptor signaling pertinent to trophoblast recognition. These actions may influence the equilibrium between physiological rebuilding of tissues and pathological inflammatory activation during implantation and early placentation [19].

Oxidative stress and metabolic dysfunction may potentially affect the functioning of decidual NK cells during recurrent reproductive failure. Reactive oxygen species accumulate and influence alterations in cytokine release, intracellular communication, mitochondrial function, and survival pathways of immune cell populations. Oxidative stress may induce a more inflammatory phenotype in decidual NK cells, which is linked to diminished trophoblast support and aberrant vascular remodeling. Vitamin D signaling seems to be intricately linked to antioxidant defense pathways and mitochondrial stability, suggesting additional mechanisms by which vitamin D may influence NK-cell activity in the implantation milieu [115].

Metabolic adaptation also influences the phenotypes of NK cells in decidual tissue. The metabolic characteristics of healthy decidual NK populations differ from those of highly cytotoxic peripheral NK cells, including variations in glucose utilization, mitochondrial function, and biosynthetic pathways. Inflammatory circumstances and metabolic stress may disrupt this unique metabolic profile and promote pro-inflammatory NK-cell activity [116]. Recent transcriptome investigations have shown the variability of decidual NK-cell populations following implantation. Distinct subsets of NK cells have unique angiogenic, immunoregulatory, inflammatory, and tissue-remodeling capabilities, influenced by their transcriptional profiles and local microenvironmental signals. Certain populations seem primarily engaged in vascular reshaping and trophoblast guidance, whereas others participate in immune control and cytokine synthesis. Imbalances among these specific subgroups may lead to implantation instability and recurrent pregnancy loss [17].

### 5.4. Macrophage Polarization and Decidual Inflammation

Macrophages constitute a significant immune-cell population inside decidual tissues, playing an active role in implantation, tissue remodeling, trophoblast invasion, vascular adaptation, and the modulation of local inflammatory responses throughout early pregnancy. Decidual macrophages have highly specialized morphologies, influenced by the hormonal, metabolic, and immunological milieu at the maternal–fetal interface, unlike circulating inflammatory macrophages which are recruited following infection or tissue damage [117]. Their activity must be regulated throughout the implantation and placentation processes, as excessive inflammatory activation or compromised tissue repair jeopardizes the progression of pregnancy from its first stages.

Physiological implantation is associated with the dynamic polarization of macrophages, rather than a homogeneous population of macrophages. Decidual macrophages exist along a functional continuum often categorized into pro-inflammatory M1-like or anti-inflammatory, tissue-remodeling M2-like phenotypes. M1 macrophages generate inflammatory cytokines, reactive oxygen species, nitric oxide, and agents of pathogen defense and tissue damage. M2-associated populations participate in extracellular matrix remodeling, angiogenesis, immunological tolerance, apoptotic cell clearance, and tissue healing [100]. During the first phases of implantation, a little inflammatory macrophage activity is essential for promoting tissue remodeling and trophoblast contact with maternal tissues. The controlled synthesis of cytokines, matrix metalloproteinases, and inflammatory mediators facilitates extracellular matrix disintegration, enhances vascular permeability, and attracts supplementary immune cell populations. During implantation, decidual macrophages gradually adopt a mostly tolerogenic and tissue-supportive phenotype that facilitates placental growth and suppresses excessive inflammatory activity. The failure of this transition leads to persistent decidual inflammation and recurrent pregnancy loss [118].

Decidual macrophages engage in ongoing communication with stromal cells, trophoblasts, uterine natural killer cells, dendritic cells, and regulatory T-cells via cytokines, chemokines, extracellular vesicles, metabolic mediators, and receptor–ligand interactions. These communication networks regulate trophoblast migration, vascular remodeling, clearance of apoptotic cells, extracellular matrix architecture, and immunological adaption [39]. Decidual macrophages play a crucial role in regulating trophoblast invasion and the formation of placental vasculature. Macrophages release angiogenic mediators such as VEGF, placental growth factor, TGF-β, and matrix-remodeling enzymes, which may affect trophoblast migration and the alteration of spiral arteries. Alterations in macrophage polarization may disrupt placental anchoring or vascular adaption during early gestation. Aberrant inflammatory signaling by macrophages may result in compromised endothelium stability and impaired placental perfusion.

Numerous investigations have shown altered macrophage polarization in women experiencing recurrent pregnancy loss. Research has revealed an elevated ratio of pro-inflammatory M1 macrophages and a diminished presence of M2 macrophages in the decidual tissue of women experiencing recurrent miscarriage. These alterations are linked to elevated production of TNF-a, IL-1b, IL-6, nitric oxide-related pathways, and oxidative mediators that contribute to tissue damage and the exacerbation of inflammation. The persistent dominance of inflammatory macrophage phenotypes may provide a hostile implantation milieu unsuitable for stable placental development [119].

Macrophage polarization in decidual tissue seems to be intricately associated with oxidative stress. Reactive oxygen species influence cytokine production, inflammatory transcription factors, mitochondrial function, and intracellular signaling pathways that impact macrophage development. Excessive oxidative stress induces inflammatory macrophage activation and increases the generation of tissue-damaging mediators. Inflammatory macrophages generate supplementary reactive oxygen species and nitric oxide, perhaps creating a self-sustaining loop of oxidative damage and decidual inflammation in cases of recurrent reproductive failure [120].

Mitochondrial metabolism is a crucial determinant of macrophage phenotype and functionality. Pro-inflammatory macrophages have distinct metabolic traits compared to tissue remodeling M2 populations, characterized by heightened glycolytic activity, modified tricarboxylic acid cycle dynamics, and enhanced inflammatory metabolic signaling. Tolerogenic macrophages mostly depend on oxidative phosphorylation and mitochondrial respiration [121]. Vitamin D signaling throughout pregnancy seems to have a growing role in the control of macrophage differentiation and inflammatory activity. Decidual macrophages possess functioning vitamin D receptors, and the activation of VDR-dependent pathways affects cytokine production, oxidative stress responses, metabolic signaling, and polarization status [74]. Vitamin D seems to downregulate inflammatory M1 activity and promote tissue-remodeling and tolerogenic macrophage phenotypes.

The influence of vitamin D on decidual macrophages may be facilitated via many molecular routes. The activation of vitamin D signaling suppresses NF-κB-dependent inflammatory transcription and reduces the production of TNF-α, IL-6, IL-1β, and other cytokines linked to M1 polarization. Simultaneously, vitamin D may enhance the expression of anti-inflammatory mediators such as IL-10 and TGF-β, as well as pathways involved in tissue remodeling and angiogenesis [122].

Vitamin D seems to alter macrophage metabolism and oxidative adaption. The activation of VDR is associated with the control of mitochondrial homeostasis, intracellular calcium signaling, antioxidant defense mechanisms, and inflammatory metabolic pathways [109]. Recent transcriptome investigations indicate that decidual macrophage populations exhibit significant variability that transcends the M1/M2 classification. Various macrophage subsets seem to be specialized for angiogenesis, extracellular matrix remodeling, immunological tolerance, clearance of apoptotic cells, or inflammatory control, contingent upon local signaling circumstances and transcriptional programming. The disruption of this precisely controlled cellular heterogeneity may hinder implantation support and lead to recurrent miscarriage via many simultaneous pathways that influence placentation and decidual stability [123].

Chronic decidual inflammation seems to be pivotal in the pathogenesis of recurrent pregnancy loss. Recurrent miscarriage may be defined by sustained low-grade inflammatory dysregulation, driven by aberrant interactions among stromal cells, macrophages, trophoblasts, oxidative stress pathways, and immunological mediators, rather than by acute, short-term inflammatory activation after implantation. In this intricate milieu, vitamin D signaling interacts with pathways that govern macrophage polarization, mitochondrial metabolism, cytokine production, and oxidative equilibrium, hence possibly serving as a significant regulator of decidual immunological homeostasis in early pregnancy [91].

### 5.5. Beyond Th17/Treg, NK Cells, and Macrophages: Emerging Immune Contributors

The maternal–fetal immune interface involves substantially more cellular diversity than the Th17/Treg axis, uterine NK cells, and macrophages discussed above. Decidual dendritic cells, particularly tolerogenic subsets expressing low costimulatory molecule levels, appear to support Treg induction and antigen-specific tolerance toward paternal alloantigens, and their dysfunction has been proposed as a contributor to implantation failure. Innate lymphoid cells, especially decidual ILC3 and ILC-like NK-cell precursor populations, have been identified as a distinct compartment from conventional uterine NK cells, with roles in tissue remodeling and cytokine production that are only beginning to be characterized in recurrent pregnancy loss. Regulatory B cells producing IL-10 and TGF-β have been described as a further tolerogenic population at the maternal–fetal interface, though their functional relevance in humans remains less established than that of Treg cells. Extracellular vesicles secreted by trophoblasts and decidual cells carry microRNAs and immunomodulatory surface proteins that may influence maternal immune cell function locally, offering a non-cellular route of immune communication. Immune checkpoint pathways, including PD-1/PD-L1 and HLA-G-mediated inhibitory signaling, have attracted particular interest as mechanisms of trophoblast immune evasion, with altered expression reported in some recurrent pregnancy loss cohorts. Single-cell transcriptomic studies of the human decidua have revealed considerably greater heterogeneity within each of these populations than bulk analyses previously suggested, and are likely to reshape the classification of decidual immune states in the coming years. Whether or how vitamin D signaling intersects with each of these newer components remains largely unexplored and represents a direction for future research rather than an established relationship.

## 6. Oxidative Stress, Mitochondrial Dysfunction, and Immunometabolism

Much of the mechanistic detail in this section is derived from cell culture models, animal studies, and a smaller body of human decidual tissue analyses. Direct clinical validation in women with recurrent pregnancy loss is available for some findings, such as altered antioxidant enzyme activity and lipid peroxidation markers, but is largely absent for others, particularly ferroptosis-specific pathways, which remain an experimental research area rather than a clinically confirmed mechanism of pregnancy loss.

### 6.1. Oxidative Stress in the Endometrial Microenvironment

Oxidative stress has become a significant component in implantation failure and recurrent pregnancy loss, especially regarding aberrant decidualization and inflammatory dysregulation. Physiological implantation requires the regulated generation of reactive oxygen species for intracellular communication, tissue remodeling, angiogenesis, and trophoblast invasion [73]. Reactive oxygen species are involved in the control of cell proliferation, cytokine signaling, extracellular matrix adaptability, and vascular transformation in the endometrium under normal circumstances. Issues occur when oxidant production surpasses the antioxidant capability of decidual tissues, leading to sustained cellular damage and impairment of pathways associated with implantation [124].

The implantation environment is especially susceptible to oxidative imbalance due to a phase of heightened metabolic and inflammatory activity. During decidual transition, stromal cells experience fast differentiation characterized by significant mitochondrial activation, elevated oxygen consumption, augmented protein synthesis, and substantial tissue remodeling. Simultaneously, trophoblast invasion, vascular adaptation, and immune cell recruitment impose additional metabolic requirements at the maternal–fetal interface [73].

Reactive oxygen species generation in decidual tissues originates from several cellular sources. Mitochondrial oxidative phosphorylation remains a significant physiological source of reactive oxygen species throughout decidualization and trophoblast function [59]. Other sources of oxidant generation are inflammatory immune cells, NADPH oxidase complexes, endoplasmic reticulum stress pathways, nitric-oxide-related indicating, and hypoxia-associated metabolic adaptation. In diseased situations, overactivation of these pathways may surpass internal antioxidant mechanisms and induce oxidative damage throughout the implantation environment [60].

Oxidative stress influences all primary facets of implantation biology. Elevated ROS levels in stromal cells lead to impaired decidual development, modified cytokine production, mitochondrial instability, reduced progesterone responsiveness, and heightened vulnerability to apoptosis. Excessive oxidative damage may also impact matrix structure and intercellular communication between decidual cells and invading trophoblasts [125]. Trophoblastic tissue demonstrates comparable sensitivity to oxidative imbalance during the first stages of placentation. Physiologic hypoxia is essential during the first stages of implantation, facilitating progressive vascular adaptation and regulated trophoblast development. Chronic oxidative stress may hinder trophoblast migration, cause mitochondrial malfunction, alter angiogenic signaling, and activate apoptotic pathways in placental tissues [126].

The correlation between oxidative stress and inflammation is particularly significant in recurrent pregnancy loss. Reactive oxygen species activate inflammatory transcription factors, including NF-κB, AP-1, and STAT-related pathways, and increase the production of cytokines such as TNF-α, IL-6, and IL-17. The continuous operation of this pathogenic cycle disrupts the decidual environment and compromises maternal–fetal immune tolerance [127].

Oxidative circumstances significantly influence immune cell populations in decidual tissues. Excessive buildup of reactive oxygen species alters macrophage polarization, enhances inflammatory dendritic cell activation, diminishes regulatory T-cell function, and promotes inflammatory natural killer cell signaling [128].

Mitochondria are crucial to these activities, serving as energy-producing organelles, regulators of intracellular transmission, and primary sources of ROS generation simultaneously. Mitochondrial malfunction may lead to aberrant ATP production, calcium dysregulation, oxidative membrane damage, activation of inflammatory pathways, and the initiation of apoptosis in decidual and trophoblastic cells [129].

Typically, antioxidant defense mechanisms protect decidual tissues from extreme oxidative damage. During implantation, metabolic activity is regulated by enzymatic pathways including superoxide dismutase, catalase, glutathione peroxidase, thioredoxin systems, and NRF2-mediated antioxidant signals to maintain intracellular redox equilibrium. A diminished antioxidant capacity has consistently been linked to recurrent pregnancy loss and implantation failure [41].

Vitamin D signaling has been progressively associated with the control of oxidative equilibrium in reproductive organs. Activation of the vitamin D receptor influences the production of antioxidant enzymes, mitochondrial homeostasis pathways, intracellular calcium signaling, and inflammatory transcription factors associated with oxidative damage. Vitamin D seems to influence NRF2-associated antioxidant responses and to restrict ROS-induced inflammatory amplification. The diminished reactivity to vitamin D is a mechanism that triggers increased oxidative stress in decidual tissues, even without significant systemic shortage [109].

The metabolic parameters of the implantation environment influence oxidative stress dynamics. During implantation, the production of reactive oxygen species and antioxidant responses are affected by hypoxia, modified glucose metabolism, mitochondrial substrate availability, and intracellular nutrient-sensing pathways. Decidual cells and trophoblasts must continuously adjust to fluctuating metabolic requirements while preserving cellular stability in relatively hypoxic environments [130].

Recent transcriptome and metabolomic investigations have progressively corroborated the idea that recurrent pregnancy loss is linked to significant oxidative and metabolic abnormalities in decidual tissues. Women with recurrent miscarriage have modified expression of antioxidant pathways, mitochondrial genes, inflammatory mediators, lipid peroxidation indicators, and oxidative-stress-response proteins. Our results indicate that oxidative imbalance is not just a subsequent consequence of implantation anomalies but may serve as a key characteristic of recurrent reproductive failure [131].

### 6.2. Mitochondrial Dysfunction in Decidual and Endometrial Cells

Mitochondria are central to endometrial physiology and decidual transformation, as implantation is among the most metabolically demanding events in human reproductive biology. Endometrial stromal cells undergo rapid differentiation during the secretory phase and early placentation, accompanied by mitochondrial biogenesis and coordinated changes in oxidative phosphorylation, glucose utilization, amino acid metabolism, and lipid turnover [43,57]. Compromised mitochondrial adaptation hinders decidual cells’ capacity to sustain implantation-supportive activity and to adjust to the rapidly changing conditions of early pregnancy [73]. Mitochondrial dynamics fusion, fission, and mitophagy are tightly regulated to preserve organelle integrity; failure of these processes leads to energy deficits, oxidative stress accumulation, and inflammatory activation within the decidual microenvironment [132]. Cells with diminished mitochondrial membrane potential, impaired ATP synthesis, or defective quality control show reduced decidual differentiation and altered expression of implantation-related genes [44].

The link between mitochondrial dysfunction and oxidative stress is particularly consequential in recurrent pregnancy loss. Impaired mitochondria generate excess reactive oxygen species that damage lipids, proteins, and mitochondrial DNA, further compromising electron transport chain activity and ATP production in a self-reinforcing cycle [133]. Mitochondrial DNA is especially vulnerable given its limited protective mechanisms relative to nuclear DNA; several studies report altered mitochondrial DNA copy number, increased damage, and abnormal mitochondrial gene expression in women with recurrent reproductive failure [134].

Several interconnected quality-control mechanisms govern this vulnerability. Mitochondria regulate intracellular calcium signaling essential for decidual development and cytoskeletal remodeling [135]; the endoplasmic reticulum–mitochondria axis coordinates calcium messaging, protein folding, and stress responses, with ER stress capable of impairing mitochondrial adaptation and prolonging inflammatory activation [136]; and autophagy/mitophagy normally clears defective mitochondria, so that impaired mitophagy allows accumulation of ROS-producing organelles and promotes apoptotic activation. Dysregulation of these autophagic pathways has been increasingly associated with recurrent reproductive failure [137].

Mitochondrial stress also shapes decidual immune function. Membrane impairment can release mitochondrial DNA and other danger-associated molecular patterns that activate inflammasome pathways, increasing IL-1β and IL-18 production and contributing to implantation instability [138]. Because tolerogenic decidual immune populations depend on oxidative phosphorylation to maintain their anti-inflammatory phenotype, this metabolic instability may favor a shift toward inflammatory, glycolytically active immune phenotypes [139].

Mitochondrial adaptability is especially important during the physiologically hypoxic conditions of early trophoblast invasion; prolonged or severe hypoxic stress can cause mitochondrial damage, oxidative imbalance, and inflammatory activation [126]. Vitamin D signaling appears to support mitochondrial function throughout this process: VDR-dependent pathways have been linked to mitochondrial biogenesis, calcium homeostasis, oxidative phosphorylation, antioxidant defense, and reduced mitochondrial ROS generation in inflammatory or oxidative environments.

Vitamin D demonstration seems to play a significant role in the control of mitochondria inside reproductive organs. VDR-dependent pathways stimulate mitochondrial biogenesis, regulate calcium homeostasis, enhance oxidative phosphorylation, bolster antioxidant defense mechanisms, and initiate intracellular stress responses. Vitamin D seems to diminish mitochondrial reactive oxygen species generation and preserve mitochondrial membrane integrity in inflammatory or oxidative environments.

### 6.3. Ferroptosis and Inflammatory Cell Death Pathways

Research on the controlled mechanisms of cell death has intensified in reproductive biology, as implantation and placental development need precise regulation of cellular survival, tissue remodeling, and inflammatory communication at the maternal–fetal interface. Physiological decidualization involves the selective elimination of damaged or senescent cells to preserve tissue homeostasis following implantation [140]. In pathological conditions, excessive activation of inflammatory cell-death pathways may undermine the integrity of decidual tissue, hinder trophoblast invasion, and exacerbate inflammatory damage during early pregnancy. Ferroptosis has been proposed as a candidate mechanism linking oxidative stress, iron metabolism, and mitochondrial dysfunction to recurrent pregnancy loss, although this remains an emerging area of research supported mainly by preclinical and experimental data [89].

Ferroptosis is an iron-dependent type of controlled cell death, marked by lipid peroxidation and severe oxidative damage to membranes. In contrast to apoptosis, which is often mediated by caspase pathways with little inflammatory response, ferroptosis is significantly linked to the exacerbation of oxidative stress, mitochondrial impairment, and inflammatory tissue injury. Ferroptotic cells acquire excessive lipid-derived reactive oxygen species, which progressively undermine cell membranes, organelles, and metabolic stability. Evidence is mounting that decidual and trophoblastic tissues may be more vulnerable to this kind of oxidative damage after implantation [88].

Various biological characteristics of the implantation environment may render decidual tissues susceptible to ferroptotic injury under pathological settings. Decidualization is linked to significant mitochondrial activation, increased oxygen consumption, accelerated cellular differentiation, and extensive inflammatory indicating, all of which render cells susceptible to oxidative stress. Early placentation relies on precisely controlled iron metabolism, angiogenesis, and hypoxia adaptation within decidual tissues. Interruption of these processes may provide circumstances conducive to unregulated lipid peroxidation and ferroptotic cell death during implantation [73,135].

Iron metabolism seems to be pivotal to this process, as intracellular iron facilitates the generation of extremely reactive oxygen radicals via Fenton chemistry. Mitochondrial respiration, DNA synthesis, and cellular proliferation after implantation need adequate physiological iron levels. Excess free intracellular iron, however, induces oxidative membrane damage and lipid peroxidation in decidual and trophoblastic cells. Modified iron metabolism, dysregulation of ferritin, and atypical intracellular iron buildup have been progressively linked to repeated reproductive failure and aberrant placental development [141].

Lipid metabolism is also implicated in vulnerability to ferroptosis. Cellular membranes include polyunsaturated fatty acids that are more susceptible to oxidative damage during elevated ROS production and compromised antioxidant defense mechanisms. During ferroptosis, oxidized phospholipids increasingly accumulate, compromising the integrity of mitochondrial, endoplasmic reticulum, and plasma membranes. During differentiation and implantation, decidual cells experience significant membrane remodeling; hence, disruptions in lipid metabolism may render cells more vulnerable to ferroptotic damage [88,90].

Glutathione peroxidase 4 serves as a principal intracellular defense mechanism against ferroptosis. GPX4, a glutathione-dependent antioxidant enzyme, mitigates oxidative membrane damage by decreasing lipid hydroperoxides. Numerous investigations have shown irregularities in glutathione metabolism and antioxidant pathways in recurrent pregnancy loss, supporting the concept that impaired redox adaption leads to implantation instability [90].

Mitochondria seem to play a significant role in ferroptotic transmission inside reproductive organs. Mitochondrial dysfunction is not only of mitochondrial origin; rather, it significantly intensifies oxidative membrane damage via elevated ROS generation, modified iron management, compromised oxidative phosphorylation, and metabolic instability. Moreover, impaired mitochondria may emit pro-inflammatory mediators and danger-associated molecular patterns that may exacerbate tissue damage in the decidual [142].

Inflammatory cytokines predispose decidual tissues to ferroptotic damage. TNF-α, IL-6, IL-1β, and IFN-γ influence stromal and trophoblastic cells, regulating iron metabolism, reactive oxygen species production, lipid remodeling, and antioxidant defense mechanisms [143]. Hypoxic stress during early placentation may also influence susceptibility to ferroptosis. Physiological hypoxia promotes trophoblast differentiation and vascular modification after implantation. Excessive or prolonged exposure to hypoxia may hinder mitochondrial adaptation, elevate ROS generation, disturb lipid metabolism, and facilitate intracellular iron buildup [126].

Recent findings indicate that ferroptosis may influence the activity of immune cells in decidual organs. Oxidative membrane damage and the production of inflammatory lipid mediators may enhance macrophage activation, cytokine secretion, and inflammatory immunological polarization at implantation sites. Vitamin D signaling seems to be more relevant in the control of ferroptosis-related pathways. VDR-dependent illustrating affects antioxidant defense mechanisms, glutathione metabolism, intracellular calcium equilibrium, inflammatory transcription factors, and mitochondrial integrity. Vitamin D seems to activate NRF2-related antioxidant mechanisms that protect against lipid peroxidation and oxidative membrane damage. Decreased response to vitamin D increases susceptibility to ferroptotic injury in the decidua during implantation [144].

The regulation of ferroptosis is linked to autophagy-related processes in reproductive organs. The selective breakdown of ferritin by ferritinophagy elevates the intracellular free iron pool and may enhance vulnerability to ferroptosis in the presence of reactive oxygen species. Recurrent pregnancy loss is associated with dysregulated autophagy and impaired mitochondrial quality control, suggesting further pathways that connect metabolic stress to ferroptotic damage during implantation [89].

### 6.4. Vitamin D and Antioxidant Defense Mechanisms

As established in Section 6.1, redox balance in the endometrium and decidua is essential for stable implantation, with excessive oxidative activity capable of rapidly disrupting the maternal–fetal interface. This section focuses specifically on the antioxidant defense mechanisms through which vitamin D signaling may help maintain that balance. Implantation depends on precisely coordinated antioxidant defense mechanisms that safeguard decidual and trophoblastic tissues from oxidative damage while preserving normal redox signaling. Growing evidence indicates the direct role of vitamin D in the control of antioxidant pathways at various cellular and molecular levels [73].

There are genomic and non-genomic influences of vitamin D transmission on intracellular oxidative equilibrium. Activation of the vitamin D receptor modulates the transcription of genes associated with the synthesis of antioxidant enzymes, mitochondrial integrity, glutathione metabolism, control of inflammation, and intracellular calcium homeostasis [145]. The NRF2 pathway is a crucial antioxidant system affected by vitamin D signaling. NRF2 serves as a principal regulator of cellular antioxidant responses by modulating detoxifying enzymes and redox-protective proteins. Under healthy settings, NRF2 activation induces the transcription of superoxide dismutase, catalase, glutathione peroxidase, heme oxygenase-1, and several other antioxidant mediators that neutralize reactive oxygen species and mitigate lipid peroxidation. Vitamin D alters NRF2-associated activity in reproductive organs, thereby enhancing endogenous antioxidant defense during implantation and placentation [42].

A significant focus of vitamin D-mediated oxidative control is the metabolism of glutathione. Glutathione is a principal intracellular antioxidant that detoxifies reactive oxygen species and protects mitochondrial membranes from oxidative damage. During decidualization, an adequate supply of glutathione is crucial, as stromal cell differentiation correlates with heightened mitochondrial activity and improved oxidative metabolism. Vitamin D transmission facilitates glutathione production and regeneration pathways, hence aiding in the maintenance of intracellular redox equilibrium within decidual tissues [146].

Vitamin D signaling further modulates the activities of superoxide dismutase and catalase. These enzymes are crucial elements of the intracellular antioxidant system, transforming highly reactive oxygen radicals into less harmful molecules prior to oxidative membrane damage. The diminished activity of these enzymes has been associated with recurrent pregnancy loss, implantation failure, and aberrant placental development. Vitamin D influences the expression and functional activity of antioxidant systems in endometrial and trophoblastic cells, indicating a protective effect against oxidative-stress-related implantation instability [147].

Another significant way by which vitamin D may maintain implantation competence is via mitochondrial protection. Mitochondria serve as the primary energy generators of the cell and the principal intracellular sources of reactive oxygen species. Excessive mitochondrial ROS generation may hinder ATP synthesis, disrupt calcium signaling, increase lipid peroxidation, and activate inflammatory or apoptotic pathways in decidual tissues. Vitamin D demonstration restores mitochondrial membrane integrity, enhances the efficiency of oxidative phosphorylation, and diminishes mitochondrial oxidative stress during inflammation [92].

The interplay between oxidative stress and inflammation is especially significant in the implantation milieu, as both systems continuously support one another. Reactive oxygen species stimulate inflammatory transcription factors, including NF-κB and MAPK-associated pathways, resulting in elevated production of TNF-α, IL-6, IL-17, and other inflammatory mediators. These cytokines subsequently stimulate ROS generation via the activation of immune cells and mitochondrial stress pathways [93]. Trophoblastic tissues significantly depend on oxidative adaption during the first stages of placentation. Extravillous trophoblasts infiltrate decidual tissues under somewhat hypoxic circumstances characterized by variable oxygen tension and considerable metabolic load. Inadequate antioxidant protection may affect trophoblast migration, angiogenic signaling, and vascular remodeling, while increasing vulnerability to apoptosis. Vitamin D improves trophoblastic antioxidant defense via modulating mitochondrial stability and intracellular stress-response mechanisms [147].

Recent data indicates that vitamin D may affect autophagy and mitophagy mechanisms that protect against oxidative cellular damage. Implantation sustains metabolic equilibrium by eliminating damaged organelles and oxidized cellular components via regulated autophagic activity. Impaired autophagy may lead to the buildup of damaged mitochondria and exacerbate oxidative stress in decidual tissues. Various processes governing autophagic adaptation and mitochondrial quality regulation under inflammatory or oxidative stress conditions seem to be associated with vitamin D activation.

## 7. Clinical Implications and Translational Perspectives

### 7.1. Vitamin D Deficiency and Clinical Reproductive Outcomes

Over the last decade, there has been a significant surge in research about the clinical ramifications of vitamin D within reproductive medicine, particularly for women experiencing recurrent pregnancy loss, implantation failure, and unexplained infertility. Historically, vitamin D was primarily evaluated for its function in skeletal metabolism; however, growing data indicates that insufficient vitamin D signaling impact implantation competence, decidual stability, placental development, and maternal immunological adaption during early pregnancy. These discoveries have led to an increasing volume of research examining vitamin D level as a potentially alterable factor linked to negative reproductive outcomes [148].

Numerous clinical investigations have shown a reduced level of circulating vitamin D in women experiencing recurrent abortion in comparison to fertile controls or women without reproductive failure. Connections between vitamin D deficiency and heightened inflammatory activity, atypical immune-cell profiles, compromised endometrial receptivity, and modified cytokine communication in decidual tissues have been documented. The findings align with the biological feasibility of a link between vitamin D dysregulation and recurrent pregnancy loss. The clinical interpretation of these data is complicated by the reality that reproductive outcomes likely rely not only on circulating vitamin D levels but also on tissue-specific response at the maternal–fetal interface [19].

A further challenge in clinical interpretation is distinguishing between systemic vitamin D insufficiency and diminished local reactivity to vitamin D in reproductive organs. Circulating blood levels may not accurately represent endometrial VDR activity, intracellular vitamin D metabolism, local CYP27B1 expression, or subsequent transcriptional response in decidual cells and immunological populations [23]. Thus, some women may have normal blood vitamin D levels but experience diminished tissue response owing to receptor malfunction, inflammatory suppression, oxidative stress, or epigenetic anomalies affecting vitamin D-related pathways.

Obesity exacerbates the clinical relationship between vitamin D and reproductive outcomes. In women, an increased BMI is often associated with decreased circulating vitamin D levels, persistent low-grade inflammatory activation, buildup of oxidative stress, insulin resistance, and modified immunological signaling. These metabolic anomalies influence implantation competence and placental development regardless of vitamin D deficiency, complicating the assessment of vitamin D shortage’s involvement. Vitamin D activation overlaps with inflammatory and metabolic pathways associated with obesity-related reproductive dysfunction, indicating potential mechanistic overlap between both disorders.

Another clinical situation in which dysregulation of vitamin D may influence implantation and pregnancy outcomes is polycystic ovarian syndrome. Vitamin D insufficiency is often seen in women with PCOS, linked to insulin resistance, chronic inflammation, oxidative imbalance, and impaired endometrial receptivity. Altered decidualization and compromised immunological adaptability have been shown in reproductive dysfunction associated with PCOS. Vitamin D regulates inflammatory announcement, mitochondrial metabolism, and progesterone sensitivity; hence, compromised vitamin D function plays a role in the intricate endometrial anomalies seen in this demographic [149].

Autoimmune illnesses linked to recurrent pregnancy loss may also influence vitamin D-related pathways. Autoimmune thyroid disease, antiphospholipid syndrome, and systemic inflammatory illnesses are often linked to persistent cytokine activation and the disruption of regulatory immune cell activity. Vitamin D affects several immunological processes, such as Treg differentiation, inflammatory cytokine synthesis, and macrophage polarization [150]. These associations raise an unresolved question of causal direction. Vitamin D deficiency frequently coexists with obesity, insulin resistance, PCOS, autoimmune thyroid disease, and chronic low-grade inflammation, each of which independently affects fertility and pregnancy outcome. It is therefore difficult, on the basis of current observational data, to establish whether low vitamin D signaling is a causal driver of implantation failure or a downstream marker of an unfavorable metabolic and immunological environment shared across these conditions. Studies that adjust for these confounders directly, or that isolate vitamin D signaling from its metabolic and autoimmune correlates, are needed before a causal role can be assigned with confidence. There is increasing interest in the correlation between vitamin D and placental vascular development. Numerous studies indicate that a shortage in vitamin D signaling impairs angiogenesis, trophoblast invasion, endothelial adaptability, and spiral artery remodeling in early pregnancy [62].

Clinical research evaluating vitamin D supplementation has shown inconclusive outcomes. Some research indicates that supplementation in deficient women increases implantation rates, strengthens immunological profiles, decreases inflammatory cytokine levels, and lowers miscarriage rates. Nevertheless, other research has shown little or inconsistent reproductive advantages. This variability likely indicates variations in research design and the biological intricacies of vitamin D communication within reproductive organs. Therapeutic response may be affected by the timing of supplementation, the severity of initial insufficiency, BMI, inflammatory condition, genetic variants influencing VDR activity, and the existence of metabolic or immunological irregularities.

### 7.2. Vitamin D Supplementation in Recurrent Pregnancy Loss and IVF

The possible therapeutic significance of vitamin D supplementation in women experiencing recurrent pregnancy loss and assisted reproductive failure has emerged as a subject of increasing clinical attention. This interest is amplified further by the increasing recognition that vitamin D is integral to various biological systems directly associated with implantation and placental development, encompassing decidual maturation, immune tolerance, oxidative regulation, mitochondrial adaptation, angiogenesis, and trophoblast communication. There is increasing interest in vitamin D supplementation as a modifiable intervention that may enhance endometrial function and implantation stability, given that several of these pathways seem to be impaired in recurrent reproductive failure [149].

Clinical research on supplementing techniques has shown varied outcomes, highlighting the biological intricacies of implantation and the many causes of recurrent miscarriage. Numerous studies indicate that rectifying vitamin D insufficiency in women correlates with enhanced clinical pregnancy rates, better implantation results, decreased miscarriage rates, and more advantageous immunological profiles. Conversely, several investigations have failed to demonstrate a definitive reproductive advantage despite the normalization of circulating blood levels [151].

A crucial factor to consider is the timing of supplementation. Vitamin D influences pathways related to implantation that start to alter during the proliferative and secretory stages of the menstrual cycle, prior to embryo transfer or conception. Gradual preparation is essential for decidual development, immune cell recruitment, cytokine modulation, and endometrial receptivity during the peri-implantation phase [19]. The duration of vitamin D exposure may also have therapeutic significance. Endometrial remodeling and immunological adaptability are dynamic biological processes necessitating continuous transcriptional and metabolic control throughout several cycles. The temporary repair of serum insufficiency does not restore decidual function or reverse persistent inflammatory and oxidative disturbances in reproductive organs. In women with chronic implantation failure or recurrent miscarriage, extended metabolic and immunological abnormalities may continue to exist despite seemingly normalized circulating vitamin D levels [152].

Numerous studies indicate that women with significant deficit may receive greater reproductive benefits from supplementing compared to those with borderline insufficiency. Severe vitamin D insufficiency correlates with heightened inflammatory activation, reduced regulatory T-cell activity, compromised decidualization, and elevated oxidative stress at implantation sites. The biological impact of rectifying these anomalies may be more pronounced in severely deficient populations than in women whose reproductive failure is primarily influenced by pathways unrelated to vitamin D transmission [74].

Supplementation may also be affected by body composition. Obesity is closely linked to chronic low-grade inflammation, insulin resistance, oxidative imbalance, and modified endometrial receptivity, as well as diminished vitamin D bioavailability. Sequestration of vitamin D in adipose tissue may diminish the availability of circulating active vitamin D, even oral supplementation. Metabolic inflammation may disrupt downstream VDR signaling in decidual tissues [153]. Genetic diversity concerning vitamin D receptor polymorphisms has been the subject of growing research in reproductive medicine. A systematic review and meta-analysis has shown that common VDR gene polymorphisms modify individual response to vitamin D supplementation, with tissue response influenced by variants affecting VDR expression, receptor binding affinity, and downstream transcriptional activity [154,155]. This gene-level variability may partly explain why supplementation trials in recurrent pregnancy loss populations have produced heterogeneous results, and represents a rationale for future genotype-stratified studies rather than a currently actionable clinical tool.

The immunological impacts of supplements seem to be more significant in women experiencing recurrent miscarriage with inflammatory or autoimmune characteristics. Certain investigations have linked vitamin D correction to heightened activity of regulatory T-cells, reduced polarization of Th17 cells, decreased production of inflammatory cytokines, and improved tolerogenic immunological profiles in reproductive organs. These modifications facilitate implantation stability by mitigating persistent inflammation of the decidua and reinstating maternal immunological tolerance to early placentation [19].

Supplementation may influence oxidative and metabolic processes associated with implantation competence. Vitamin D is associated with enhanced mitochondrial integrity, increased antioxidant enzyme activity, reduced ROS generation, and regulation of inflammatory oxidative signaling. Considering that oxidative stress and mitochondrial dysfunction are significant contributors to recurrent reproductive failure, the enhancement of cellular redox equilibrium may be a mechanism via which vitamin D promotes implantation and early placental development [156].

The use of vitamin D in assisted reproductive technologies is particularly contentious. An appropriate vitamin D level has been linked to enhanced embryo transfer outcomes, increased implantation rates, or better clinical pregnancy rates in several IVF trials. Other studies have failed to establish any meaningful correlation between blood vitamin D levels and IVF success. The discrepancies in results are likely attributable to variations in stimulation procedures, embryo quality, patient age, BMI, embryo stage at transfer, and endometrial pathology [157].

Taken together, randomized controlled trials and meta-analyses of vitamin D supplementation in reproductive populations have produced heterogeneous and, at times, contradictory results, and no major reproductive medicine guideline currently recommends vitamin D supplementation specifically for the prevention of recurrent pregnancy loss. Correction of documented vitamin D deficiency remains appropriate for general maternal health, but this should not be conflated with a dedicated RPL-prevention indication, which current evidence does not yet support.

Interpreting vitamin D data in IVF requires caution when differentiating between embryo competency and endometrial receptivity, particularly given that randomized trials of vitamin D supplementation in IVF populations have produced inconsistent results and do not currently support a personalized dosing strategy based on reproductive outcome alone. Implantation failure may arise from embryonic chromosomal anomalies, inadequate endometrial receptivity, modified immunological communication, disrupted decidualization, or concurrent pathologies affecting many systems. The significance of vitamin D in endometrial and immunological processes may surpass its impact on embryonic developmental competence, thereby explaining why its advantages are more evident in certain implantation studies compared to overall IVF result studies [158].

### 7.3. Translational Biomarkers and Personalized Reproductive Medicine

It should be stated clearly that none of the biomarkers discussed in this section, including endometrial VDR expression, CYP27B1 activity, transcriptomic signatures, or extracellular vesicle profiles, are currently validated for clinical use or incorporated into routine recurrent pregnancy loss evaluation. No standardized assay, reference range, or diagnostic threshold presently exists for tissue-level vitamin D signaling, and there is currently insufficient evidence that measuring it changes diagnosis or treatment. The discussion that follows describes research directions and biological rationale, not current practice recommendations.

As the comprehension of recurrent pregnancy loss as a condition characterized by interrelated immunological, metabolic, oxidative, and decidual anomalies has advanced, there has been a heightened interest in creating translational biomarkers to discern the underlying pathological mechanisms in individual patients [159]. The standard diagnostic evaluation for recurrent miscarriage often includes anatomical examination, endocrine testing, thrombophilia screening, and parental karyotyping. Notwithstanding this research, a considerable percentage of women continue to be categorized as experiencing unexplained reproductive failure. This ‘unexplained’ category increasingly reflects the limitations of current clinical evaluation rather than the lack of biological malfunction.

The implantation process involves specific molecular interactions that transpire within a limited time framework, governed by dynamic communication among decidual stromal cells, trophoblasts, immune cell populations, vascular architecture, and metabolic pathways. Disruption of these mechanisms may be clinically asymptomatic but may significantly impact cellular implantation capability. Translational biomarkers promise to transition from descriptive clinical classifications to mechanistically defined reproductive phenotypes that might inform tailored therapy methods [160].

Endometrial transcriptome profiling is one of the most promising methodologies in this domain. High-throughput sequencing and molecular expression analyses have revealed specific gene signatures related to implantation that are linked to recurrent pregnancy loss, recurrent implantation failure, faulty decidualization, inflammatory activation, and compromised immunological tolerance. Decidual tissues from women experiencing reproductive failure consistently exhibit altered expression of genes associated with progesterone responsiveness, cytokine signaling, oxidative defense, angiogenesis, extracellular matrix remodeling, and vitamin D-related pathways [161].

There is considerable interest in molecular markers of decidualization, as a failure in stromal cell differentiation seems to be pivotal to implantation instability. Anomalous endometrial receptivity during the implantation window correlates with reduced expression of prolactin, IGFBP-1, FOXO1, HOXA10, HAND2, and adhesion molecules pertinent to implantation. The findings suggest that molecular evaluation of decidual competence may provide therapeutically significant insights beyond traditional histological analysis or blood hormone levels [162].

The immune profile of the endometrium has received significant interest. Recurrent pregnancy loss is linked to aberrant regulatory T-cell function, excessive Th17 polarization, modified macrophage morphologies, heightened NK-cell activation, and an imbalance of inflammatory cytokines. The measurement of cytokines, including IL-6, IL-10, TNF-α, IL-17, and TGF-β, with immune cell characterization, facilitate the identification of inflammatory decidual phenotypes associated with compromised maternal–fetal tolerance. Such methodologies may potentially enable more personalized immunomodulatory therapy regimens for certain individuals [163].

A possible future translational avenue involves biomarkers associated with vitamin D, though this remains investigational. Contemporary clinical practice relies exclusively on blood 25-hydroxyvitamin D levels; tissue-level assessment of endometrial vitamin D signaling is not part of current clinical care, and these circulating concentrations may not correctly represent tissue-specific reactivity in reproductive organs.

Biological activity may be affected by variables outside blood levels, including local VDR expression, CYP27B1 activity, downstream transcriptional responses, inflammatory suppression of vitamin D communication, and receptor polymorphisms. Future biomarker efforts may need a combined evaluation of systemic and endometrial vitamin D pathways, rather than only relying on serum levels [155].

Genetic and epigenetic profiling may enhance our comprehension of recurrent reproductive failure. Implantation instability and recurrent miscarriage have been progressively associated with VDR signaling, inflammatory pathways, oxidative defense mechanisms, angiogenesis, and decidual transcriptional control. Simultaneously, changes in DNA methylation patterns, histone modifications, chromatin remodeling, and non-coding RNA expression have been seen in decidual tissues from women experiencing reproductive failure. According to these results, endometrial cellular programming anomalies remain across many cycles, which might explain why some women have repeated miscarriages [164].

MicroRNAs and extracellular vesicles have emerged as compelling potential for biomarkers due to their involvement in facilitating intercellular communication during implantation. Decidual cells, trophoblasts, immune cells, and endothelial tissues secrete extracellular vesicles containing microRNAs that may modulate inflammatory signaling, angiogenesis, oxidative equilibrium, mitochondrial adaptability, and trophoblast activity. Changes in extracellular vesicle cargo have been shown in reproductive diseases such as recurrent pregnancy loss and implantation failure [165].

Interest in mitochondrial biomarkers is also on the rise. Alterations in mitochondrial DNA copy number, expression of oxidative phosphorylation genes, cytoplasmic buildup of ROS, and signs of oxidative membrane damage have been linked to recurrent reproductive failure. Given that implantation requires considerable metabolic adaption in the decidual and trophoblastic tissues, evaluating mitochondrial activity may provide critical insights into implantation competence and placental stability. Another significant domain of translation is to indicators of oxidative stress and antioxidant capability. Women experiencing recurrent miscarriage have higher lipid peroxidation products, modified glutathione metabolism, diminished antioxidant enzyme activity, and heightened oxidative DNA damage. The comprehensive assessment of oxidative and inflammatory indicators assists in identifying individuals for whom oxidative damage significantly contributes to implantation instability and recurrent pregnancy loss [166].

Recent advancements in single-cell transcriptomics have revolutionized the comprehension of implantation biology, revealing significant cellular variability among decidual tissues. Various stromal, immunological, endothelial, and trophoblastic subpopulations possess distinct transcriptional programs linked to implantation support, angiogenesis, inflammatory control, or stress responses [167]. The disruption of equilibrium among these cellular states directly leads to reproductive failure. Single-cell methodologies may enable the future detection of extremely precise pathogenic implantation signals in particular patients. Artificial intelligence and machine learning techniques are being used to integrate complicated biological datasets into therapeutically useful prediction models. Recurrent pregnancy loss is probably attributable to the combined failure of several interacting systems rather than isolated abnormalities of specific biomarkers.

## 8. Discussion

Recurrent pregnancy loss is now acknowledged as a complex problem arising from impaired maternal–fetal adaptation during implantation and early placental development, rather than being only attributed to embryonic chromosomal abnormalities or isolated endocrine dysfunction. Recurrent miscarriage correlates with persistent inflammatory activation, impaired maternal immune tolerance, and irregular interactions among decidual immune cells within the implantation microenvironment.

Ota et al. showed that vitamin D signaling facilitates the development and stability of FoxP3-positive regulatory T-cells [168]. Qin et al. documented heightened Th17-mediated inflammatory responses, characterized by elevated IL-17, TNF-α, and IL-6 expression in women experiencing recurrent pregnancy loss [169]. Yan et al. found that decreased vitamin D levels were linked to modified maternal immunological tolerance pathways during early pregnancy [72]. Furthermore, Farazmand et al. validated the correlation between vitamin D deficiency and persistent inflammatory decidual activation [170]. Taken together, these studies are consistent with the hypothesis that impaired vitamin D signaling contributes to a failure of the physiological transition from implantation-related inflammation to sustained maternal immunological tolerance, although the evidence remains predominantly observational and mechanistic rather than causal.

The link between vitamin D transmission and decidual immune adaptability seems to be much more intricate than just a correlation between serum insufficiency and inflammatory activation. Ota et al. demonstrated that vitamin D directly influences Treg differentiation and suppressive stability by regulating FoxP3-associated transcriptional pathways, indicating that enough vitamin D activity may aid in sustaining tolerogenic immunological communication during implantation [168]. Qin et al. concurrently established that recurrent miscarriage in women correlates with persistent activation of Th17-related inflammatory pathways, which may result in heightened oxidative damage and hindered trophoblast invasion [169]. When combined with the results of Yan et al. and Farazmand et al., which linked low vitamin D activity to a lack of immunological adaption in the mother during the first trimester of pregnancy, these findings take on further significance [72,170]. The evidence increasingly indicates that vitamin D deficiency may contribute to persistent low-grade inflammatory instability in decidual tissues, rather than only serving as a biomarker of accidental biochemical abnormalities.

The existing findings are consistent with faulty decidualization playing a significant role in recurrent pregnancy loss. Li et al., Guo et al., and Du et al. have collectively demonstrated that vitamin D signaling modulates stromal cell differentiation, transcriptional pathways associated with implantation, progesterone responsiveness, and endometrial receptivity during the peri-implantation phase [12,171,172]. Li et al. reported that vitamin D modulates the expression of prolactin and IGFBP-1 in decidual stromal cells, suggesting a role for vitamin D signaling in decidual maturation [172]. Du et al. discovered that vitamin D signaling also promoted regulation of implantation-related genes such as HOXA10 and adhesion-associated pathways in human endometrial tissues [171]. Guo et al. expanded these findings by discovering modified VDR expression in women experiencing repeated reproductive failure, indicating that compromised local tissue response may significantly contribute to implantation instability, regardless of blood vitamin D levels alone [12].

Kuroshli et al. (2024) and Guo et al. (2020) further substantiated this notion by demonstrating that vitamin D is intricately associated with pathways pertinent to endometrial receptivity, progesterone responsiveness, and the transcriptional control of implantation during the secretory phase [9,12]. Their results are compatible with the role of vitamin D in supporting the development of a receptive decidual phenotype conducive to trophoblast invasion and maternal immunological tolerance, though a direct causal contribution has not been established in humans. Du et al. (2005) demonstrated that inflammatory activation and oxidative stress disrupt progesterone-dependent decidual signaling pathways in populations experiencing recurrent miscarriage [171]. The findings indicate that prolonged decidual inflammation may diminish stromal cell response despite sufficient endocrine support.

The correlation between vitamin D activation and progesterone responsiveness is particularly significant, as many women with recurrent miscarriages have normal blood progesterone levels, despite molecular evidence indicating compromised decidual transformation [7]. The interplay between progesterone-mediated transcriptional pathways and vitamin D-regulated communication networks is essential for effective decidualization, as shown by Guo et al. (2020) [12]. Kuroshli et al. (2024) subsequently illustrated the linkages between vitamin D activity and the pathways of endometrial receptivity during the implantation window [9]. Guo et al. (2020) showed that vitamin D transmission directly modulates indicators related with decidualization [12]. Gellersen et al. (2014) demonstrated that inflammatory and oxidative stress pathways diminish downstream progesterone response in decidual tissues [173].

Immune dysregulation, including uterine natural killer cells and macrophage polarization, was regularly highlighted in the literature. Moffett et al. (2016), Fraser et al. (2012), and Robson et al. (2012) emphasized the physiological function of decidual NK cells in directing trophoblasts and remodeling spiral arteries [48,174,175]. Fu et al. (2017) further demonstrated the significance of decidual immune cell-mediated vascular adaptation for stable placental development [176]. Simultaneously, Houser (2012) and Zhang et al. (2017) demonstrated that appropriate decidual macrophage polarization is essential for successful implantation and placental development [100,177]. Disruption of the M1/M2 balance and excessive inflammatory decidual signaling, by contrast, contribute to implantation failure and recurrent pregnancy loss.

The significance of these results is emphasized by research indicating that vitamin D signaling is a crucial regulator of the immunological milieu at the maternal–fetal interface. Ota et al. (2014) indicated that vitamin D insufficiency in women experiencing recurrent pregnancy loss correlates with heightened cellular immunity and decreased immunological tolerance [168]. Abdollahi et al. (2020) demonstrated that 1,25(OH)2D3 regulates the expression of FOXP3, RORγt, and significant cytokines pertinent to the Treg/Th17 equilibrium [178]. Zhao et al. (2021) performed an extensive assessment of the current information regarding vitamin D insufficiency and its correlation with dysregulated decidual immunity, modified cytokine production, and impaired implantation [150]. Collectively, these data indicate that vitamin D influences implantation competence via complex interactions among decidual macrophages, uterine natural killer cells, regulatory T-cells, and cytokine signaling networks, while also mitigating inflammation. The disruption of integrated immunological pathways may ultimately affect trophoblast invasion, spiral artery remodeling, and maternal–fetal immune tolerance during early placentation.

Oxidative stress and metabolic dysfunction have consistently been identified as significant contributors to recurrent pregnancy loss in the research reviewed. The excessive formation of ROS, compromised antioxidant defenses, mitochondrial dysfunction, and disturbed cellular bioenergetics provide an adverse environment for implantation and early placental development (Table 2). Agarwal et al. (2012) and Ruder et al. (2009) have shown that women experiencing reproductive failure frequently display heightened oxidative stress and diminished antioxidant capacity, thereby supporting the hypothesis that redox status dysregulation may impair endometrial function and the maintenance of pregnancy [110,127]. Similarly, Van Blerkom (2011) and May-Panloup et al. (2016) underscored the significance of mitochondrial integrity and energy metabolism for reproductive competence [179,180]. Normal cellular activity during the initial phases of embryo implantation and placental development relies on sufficient mitochondrial function [179,180].

Additional data indicates that oxidative stress, inflammatory signaling, and compromised decidual function are strongly interconnected in recurrent pregnancy loss. Women experiencing recurrent miscarriage had markedly increased oxidative stress indicators and reduced antioxidant capacity, as shown by Zejnullahu et al. (2021), suggesting that persistent redox imbalance may contribute to pregnancy failure [166]. Gonçalves et al. (2018) highlighted the possible role of vitamin D deficiency in inducing inflammatory and oxidative dysfunctions at the maternal–fetal interface, which may generate an adverse environment for implantation and early placental development [181]. Sun et al. (2023) recently reported that ferroptosis-related lipid peroxidation and iron-dependent oxidative damage in decidual stromal cells may contribute to recurrent pregnancy loss [141].

A prevalent topic in the research was the strong association between vitamin D signaling and trophoblast biology. Research conducted by Barrera et al. (2015) and Liu et al. (2011) has shown that vitamin D modulates various processes integral to placental development, encompassing trophoblast migration, invasion, angiogenesis, immune modulation, and interaction with the decidual microenvironment [182,183]. Chan et al. (2015) demonstrated that vitamin D improves extravillous trophoblast invasion, whereas Kim et al. (2018) indicated that vitamin D aids trophoblast migration via processes associated with epithelial–mesenchymal transition [45,184]. Liu et al. (2011) concurrently established that vitamin D regulates inflammatory signaling and angiogenic pathways in the placenta, therefore fostering an environment conducive to proper implantation and placental differentiation [183].

Recent research further substantiates the idea that vitamin D dysregulation may influence placental adaptation primarily via immunological mechanisms rather than only via trophoblast invasion. Yue et al. (2024) indicated that vitamin D shortage linked to CYP27B1 was correlated with impaired CD4+ T-cell function in recurrent spontaneous miscarriage, suggesting a relationship between abnormal vitamin D metabolism and disrupted maternal immunological tolerance [46]. Research on decidual natural killer cells has shown that immune-mediated vascular remodeling is essential for proper placentation. Robson et al. (2012) discovered that uterine NK cells are involved in the first phase of spiral artery remodeling, whereas Fu et al. (2017) noted that certain subsets of decidual NK cells support fetal development via the secretion of growth-promoting substances [48,176].

Studies investigating vitamin D supplementation in women with recurrent reproductive failure have generally shown varied clinical results, while numerous similar molecular processes have been identified. Vitamin D insufficiency correlates with heightened cellular immunity and autoimmunity in women experiencing recurrent pregnancy loss; restoring sufficient vitamin D levels may aid in re-establishing immunological tolerance at the maternal–fetal interface. Abdollahi et al. (2020) showed that 1,25-dihydroxyvitamin D3 therapy affects the expression of FOXP3, RORγt, and other immune-related markers associated with the Treg/Th17 axis, hence confirming its immunoregulatory function during early pregnancy [178]. Despite these mechanistic results, observational investigations of women undergoing in vitro fertilization have shown contradictory correlations between circulating vitamin D levels and implantation outcomes. The discrepancy may arise from the greater significance of local endometrial response compared to blood vitamin D levels alone. Guo et al. (2020) demonstrated the dynamic expression of VDR and CYP27B1 in the human endometrium during the menstrual cycle, suggesting that tissue-specific vitamin D signaling may significantly influence endometrial receptivity and implantation competence [12].

Vitamin D signaling should be interpreted as one contributor among several interacting mechanisms in recurrent pregnancy loss, not a dominant explanation for unexplained cases. Chromosomal abnormalities, endocrine disorders, uterine anomalies, antiphospholipid syndrome, inherited thrombophilia, chronic endometritis, embryonic factors, and broader genetic susceptibility remain established and, in many cohorts, are more frequently identified causes. The immunometabolic pathways discussed here are best viewed as one piece of a multifactorial disease process, and in many patients, vitamin D dysregulation likely coexists with, rather than substitutes for, these other mechanisms.

An additional immune population meriting attention in this context is myeloid-derived suppressor cells (MDSCs). In a mouse pregnancy model, depletion of MDSCs increased the cytotoxicity of decidual natural killer cells and precipitated pregnancy loss, supporting a protective role for MDSCs in maintaining feto-maternal immune tolerance [185]. Whether vitamin D signaling modulates MDSC function in the reproductive context specifically has not been directly studied, but evidence from tumor immunology indicates that VDR is expressed in MDSCs and that 1,25-dihydroxyvitamin D can inhibit their T-cell suppressive function and reduce nitric oxide synthase production [186]. This raises the possibility, not yet tested in a reproductive setting, that vitamin D could influence MDSC-mediated tolerance at the maternal–fetal interface, representing a further avenue for future investigation alongside the other emerging immune contributors discussed above.

## 9. Conclusions

Recurrent pregnancy loss remains one of the most complex biological disorders of reproductive medicine. Healthy implantation and placentation require optimal synchronization of decidual transformation, immune tolerance, oxidative homeostasis, mitochondrial adaptation, trophoblast invasion, angiogenesis and inflammatory regulation at the maternal–fetal interface. The evidence presented in this review strongly supports the notion that vitamin D signaling is directly involved in many of these pathways at the same time, far beyond its classical endocrine role in calcium metabolism. The existing literature suggests that impaired vitamin D activity may contribute to implantation instability and early pregnancy failure by modulating the immunometabolic networks required for maternal adaptation in early gestation.

The literature reviewed consistently demonstrated the effect of vitamin D on critical biological processes involved in implantation competence. These include decidual stromal-cell differentiation, progesterone responsiveness, Treg stabilization, Th17 suppression, and cytokine regulation, as well as macrophage polarization, uterine natural killer cell activity, trophoblast invasion, mitochondrial homeostasis, and antioxidant defense mechanisms. Crucially, it seems that recurrent pregnancy loss is characterized by the simultaneous dysregulation of many of these systems rather than the isolated dysfunction of a single pathway. These observations may provide some insight into why the conventional diagnostic approach often fails to elucidate a clear etiology in a significant proportion of women who present with recurrent miscarriage.

It is concluded that serum vitamin D levels may not be sufficient to reflect the responsiveness of reproductive tissues alone. Multiple studies have highlighted local VDR expression, decidual vitamin D metabolism, inflammatory inhibition of receptor signaling, oxidative stress pathways and tissue-specific transcriptional regulation within the implantation environment. Future reproductive assessment may therefore need to extend beyond the simple biochemical classification of vitamin D deficiency and towards more detailed characterization of endometrial vitamin D responsiveness and its interaction with inflammatory and metabolic pathways involved in implantation.

Clinical supplementation studies have produced heterogeneous results, but the inconsistencies in the literature are likely to reflect the biological heterogeneity of recurrent reproductive failure itself. Women with inflammatory decidual phenotypes, immune tolerance deficiencies, susceptibilities to oxidative stress, mitochondrial dysfunction or defective VDR signaling may respond differentially to vitamin D-based interventions than women whose pregnancy losses are predominantly caused by unrelated mechanisms. These findings underscore the need to develop more personalized and mechanistically based therapeutic approaches in reproductive medicine, though it should be stated plainly that no such stratified approach currently exists in validated form, and present evidence does not support vitamin D supplementation as a standard intervention for recurrent pregnancy loss outside the correction of documented deficiency.

Future studies should seek to characterize implantation-specific reproductive phenotypes by using integrated translational models comprising endometrial transcriptomics, immune profiling, oxidative stress biomarkers, mitochondrial assessment, extracellular vesicle signaling and vitamin D-related molecular pathways. The identification of women with impaired immunometabolic adaptation during implantation may allow for a better stratification of patients and more targeted interventions to restore the decidual competence and maternal immune tolerance.

In conclusion, vitamin D seems to be a key immunometabolic regulator in the implantation niche, modulating a number of biological systems that are crucial for the establishment of a successful pregnancy. Disorders of vitamin D signaling may play a major role in recurrent pregnancy loss via combined effects on decidualization, inflammation regulation, oxidative balance, mitochondrial stability, trophoblast invasion and placental development. Further integration of molecular reproductive biology and personalized clinical medicine will likely be needed to advance research in this area, though translation of these mechanistic insights into validated clinical management strategies remains a task for future studies rather than a present-day capability.

## Figures and Tables

**Table 1:** Molecular and cellular actions of Vitamin D at the maternal–fetal interface.

Biological Process	Main Cellular Targets	Principal Molecular Mediators	Effects of Vitamin D Signaling	Potential Clinical Relevance	Principal Level of Evidence
Decidualization. [11,33]	Endometrial stromal cells	VDR, PR, HOXA10, HAND2, prolactin, IGFBP1	Enhances stromal differentiation, progesterone responsiveness, and receptivity-related transcription	Impaired signaling may contribute to implantation instability	Human decidual tissue + in vitro
Endometrial receptivity [12,14]	Luminal and glandular epithelium	Integrins, LIF, HOXA10, cytokine pathways	Supports receptive endometrial phenotype during the implantation window	Potential biomarker of implantation competence	Human endometrial biopsy studies
Regulatory immune adaptation [34,35]	Regulatory T-cells, dendritic cells	FoxP3, IL-10, TGF-β	Promotes maternal immune tolerance and suppressive immune signaling	Potential target for immunomodulatory therapy	Human + animal model
Th17-associated inflammation [36,37]	Th17 cells, macrophages	IL-17, STAT3, RORγt, TNF-α	Suppresses inflammatory polarization and cytokine amplification	Predictor of inflammatory reproductive phenotype	Human decidual/peripheral studies
Decidual immune-cell communication [38,39]	NK cells, macrophages, trophoblast	HLA-G, VEGF, immune checkpoint pathways	Regulates trophoblast–immune interaction and vascular adaptation	Associated with placental stability	In vitro + limited human tissue
Cytokine regulation [19,40]	Decidua, trophoblast, immune cells	NF-κB, IL-6, TNF-α, IFN-γ	Reduces excessive inflammatory signaling	May influence miscarriage susceptibility	Human + in vitro
Oxidative stress defense [41,42]	Decidual and trophoblast cells	NRF2, glutathione, SOD, catalase	Enhances antioxidant defense and intracellular redox balance	Protection against oxidative implantation injury	In vitro + animal model
Mitochondrial adaptation [43,44]	Stromal cells, trophoblast	Oxidative phosphorylation, ATP production, calcium signaling	Preserves mitochondrial stability and metabolic flexibility	Potential role in implantation resilience	In vitro + animal model
Trophoblast invasion [45,46]	Extravillous trophoblast	MMP-2, MMP-9, VEGF, ERK signaling	Promotes migration, invasion, and placental anchoring	Important for early placental development	In vitro (trophoblast cell lines)
Angiogenesis and vascular remodeling [47,48]	Decidua, endothelial cells	VEGF, angiopoietins, placental growth factors	Supports spiral artery remodeling and placental perfusion	May affect placental insufficiency risk	Human placental tissue + animal model

Vitamin D indicating modulates a spectrum of interconnected pathways that are implicated in implantation, decidualization, immune tolerance, trophoblast invasion, oxidative homeostasis, mitochondrial adaptation, and placental development. Disruption of these pathways may lead to recurrent pregnancy loss through immunological and metabolic mechanisms. Abbreviations: VDR, vitamin D receptor; PR, progesterone receptor; HAND2, heart and neural crest derivatives expressed 2; IGFBP1, insulin-like growth factor binding protein 1; LIF, leukemia inhibitory factor; FoxP3, forkhead box P3; TGF-β, transforming growth factor beta; STAT3, signal transducer and activator of transcription 3; RORγt, retinoic acid receptor-related orphan receptor gamma t; TNF-α, tumor necrosis factor alpha; HLA-G, human leukocyte antigen G; VEGF, vascular endothelial growth factor; NF-κB, nuclear factor kappa B; IFN-γ, interferon gamma; NRF2, nuclear factor erythroid 2-related factor 2; SOD, superoxide dismutase; MMP, matrix metalloproteinase; ERK, extracellular signal-regulated kinase.

**Table 2:** Integrated immunometabolic network potentially linking vitamin D dysregulation with recurrent pregnancy loss.

Pathophysiological Mechanism	Cellular and Molecular Features	Biological Consequences	Potential Translational Relevance	Potential Relevance of Vitamin D
Defective decidualization [11,33]	Impaired stromal differentiation, altered HOXA10 and IGFBP1 signaling	Reduced implantation competence	Marker of endometrial dysfunction	VDR activation supports stromal differentiation and progesterone-responsive gene expression
Chronic inflammatory activation [19,40]	Persistent TNF-α, IL-6, IL-17 signaling	Decidual instability and trophoblast injury	Potential target for anti-inflammatory therapy	Suppresses NF-κB-dependent cytokine production
Treg/Th17 disequilibrium [34,36]	Reduced immune tolerance and exaggerated inflammatory polarization	Maternal–fetal immune imbalance	Immune profiling candidate	Promotes Treg differentiation and suppresses Th17 polarization
Oxidative stress accumulation [41,60]	ROS-mediated mitochondrial and membrane injury	Cellular dysfunction and inflammatory amplification	Oxidative stress biomarker	Enhances NRF2-mediated antioxidant defense
Mitochondrial dysfunction [43,44]	Impaired oxidative phosphorylation and ATP production	Reduced metabolic adaptation during implantation	Target for metabolic intervention	Supports mitochondrial biogenesis and calcium homeostasis
Ferroptosis-related lipid peroxidation [89,90]	Iron-dependent oxidative membrane damage	Inflammatory decidual injury	Emerging molecular target	May reduce ferroptotic vulnerability via antioxidant and iron-regulatory pathways
Impaired trophoblast invasion [45,46]	Reduced MMP signaling and migratory capacity	Defective placental anchoring	Predictor of placental dysfunction	Modulates MMP-2/9 and VEGF signaling relevant to invasion
Abnormal angiogenesis [47,48]	Impaired VEGF-related vascular remodeling	Reduced placental perfusion	Potential marker of placental insufficiency	Regulates VEGF and angiopoietin expression
Dysregulated immune-cell communication [38,91]	Abnormal NK-cell and macrophage signaling	Defective implantation microenvironment	Translational immunological target	Modulates NK-cell and macrophage polarization toward tolerogenic phenotypes
Impaired local vitamin D signaling [9,12]	Altered VDR/CYP27B1-related pathways	Reduced tissue responsiveness despite normal serum levels	Candidate precision-medicine biomarker	Directly reflects VDR/CYP27B1 dysfunction; correction may require tissue-level rather than serum-level intervention
Oxidative-inflammatory feedback loops [92,93]	ROS-driven NF-κB activation and cytokine amplification	Persistent decidual inflammation	Mechanistic therapeutic target	May interrupt this loop via simultaneous NF-κB suppression and antioxidant enhancement
Metabolic inflammatory adaptation failure [43,92]	Combined mitochondrial, oxidative, and inflammatory dysregulation	Implantation instability and pregnancy loss	Basis for immunometabolic stratification	Combined effects on mitochondrial, oxidative, and inflammatory pathways position it as a potential multitarget modulator

**Table 3:** Immunological abnormalities in recurrent pregnancy loss and potential modulatory effects of vitamin D.

Immunological Alteration in RPL	Main Biological Features	Molecular Pathways Involved	Potential Effects of Vitamin D	Translational Implications
Reduced regulatory T-cell activity [34,35]	Impaired maternal–fetal tolerance	FoxP3, IL-10, TGF-β	Promotes Treg differentiation and functional stability	Candidate biomarker of immune tolerance
Increased Th17 polarization [36,37]	Chronic decidual inflammation	IL-17, STAT3, RORγt	Suppresses Th17-associated inflammatory signaling	Potential target for immune stratification
Elevated pro-inflammatory cytokines [19,40]	Excessive inflammatory activation	TNF-α, IL-6, NF-κB	Reduces cytokine amplification and inflammatory transcription	Associated with implantation instability
Abnormal uterine NK-cell activation [38,39]	Defective trophoblast guidance and vascular remodeling	VEGF, IFN-γ, HLA-C/HLA-G interactions	Modulates NK-cell inflammatory behavior	Relevant to placental adaptation
Pro-inflammatory macrophage predominance [91,100]	Persistent inflammatory decidual phenotype	M1/M2 polarization, IL-1β, TNF-α	Supports tolerogenic macrophage polarization	May influence chronic decidual inflammation
Impaired immune checkpoint signaling [39,110]	Defective maternal immune adaptation	PD-1/PD-L1, HLA-G, CTLA-4	May support tolerogenic immune pathways	Emerging immunological target
Oxidative-inflammatory amplification [92,93]	ROS-mediated tissue injury	NF-κB, mitochondrial ROS, inflammatory cytokines	Enhances antioxidant defense systems	Potential marker of oxidative reproductive stress
Impaired trophoblast–immune interaction [37,47]	Defective placental establishment	Cytokine imbalance, angiogenic disruption	Supports trophoblast communication and angiogenesis	May influence early placental failure
Aberrant dendritic-cell activation (82]	Excessive antigen presentation and immune activation	IL-12, antigen-presenting pathways	Promotes tolerogenic dendritic-cell phenotype	Potential mechanism of immune-mediated RPL
Persistent decidual inflammation [19,40]	Chronic inflammatory microenvironment	IL-6, IL-17, TNF-α, oxidative pathways	Limits inflammatory amplification	Associated with recurrent implantation instability

## Data Availability

No new data were created or analyzed in this study. Data sharing is not applicable to this article.
